# aDISCO: A clearing method to enable 3D microscopy of large archival paraffin-embedded human tissue blocks

**DOI:** 10.1126/sciadv.aef9926

**Published:** 2026-08-12

**Authors:** Anna Maria Reuss, Dominik Groos, Martina Cerisoli, Lena Nordberg, Lukas Frick, Fabian F. Voigt, Nikita Vladimirov, Philipp Bethge, Regina Reimann, Fritjof Helmchen, Peter Rupprecht, Adriano Aguzzi

**Affiliations:** ^1^ Institute of Neuropathology, University Hospital Zurich, Schmelzbergstrasse 12, 8091 Zurich, Switzerland.; ^2^ Brain Research Institute, University of Zurich, Winterthurerstrasse 190, 8057 Zurich, Switzerland.; ^3^ Neuroscience Center Zurich, University of Zurich, Winterthurerstrasse 190, 8057 Zurich, Switzerland.; ^4^ University Research Priority Program (URPP), Adaptive Brain Circuits in Development and Learning, University of Zurich, Zurich, Switzerland.; ^5^ Center for Microscopy and Image Analysis (ZMB), University of Zurich, Winterthurerstrasse 190, 8057 Zurich, Switzerland.; ^6^ Institute for the Science of the Aging Brain (ISAB), Lerchenfeldstrasse 3, 9014 St. Gallen, Switzerland.

## Abstract

Human surgery and autopsy specimens are routinely stored as formalin-fixed paraffin-embedded (FFPE) tissue blocks for decades, creating vast archives of healthy and diseased tissues. While tissue clearing and whole-mount microscopy enable 3D analysis, FFPE human tissue blocks are often unsuitable for clearing and immunolabeling due to their large size and extensive cross-linking. Here, we introduce “archival” DISCO (aDISCO), a clearing method designed to overcome these challenges. aDISCO achieves effective clearing and immunolabeling of large samples stored for 15 years or more. We applied aDISCO to human brain, spinal cord, peripheral nerve, skin, muscle, heart, kidney, liver, spleen, colon, and lung, using a broad range of antibodies. Combining aDISCO with deep learning–based analysis to study focal cortical dysplasia (FCD), we found disrupted cortical layering with both focal and global neuronal density variations, features likely to be overlooked by conventional histology. In summary, aDISCO delivers datasets suitable for deep learning–based processing, enabling the detection of subtle and sparse pathologies in large archival human tissue specimens.

## INTRODUCTION

Histology is an ubiquitous tool for the diagnosis of diseases and for biological research (*1*, *2*). Formalin-fixed, paraffin-embedded (FFPE) tissue preservation was pioneered by F. Blum in 1893 (*3*) and became widely adopted in the early 20th century. FFPE tissue blocks are usually sliced into thin sections measuring about 5 μm (*4*). FFPE histology generates optically clear sections accessible to transmission light microscopy. However, these analyses are intrinsically two-dimensional (2D) and enable the examination of only a small fraction of the tissue specimen. This, in turn, may prevent the detection of important pathologies and can only partially be obviated by the laborious scrutiny of numerous layered sections. These limitations impair the evaluation of spatially complex, convoluted structures such as blood vessels and nerve fibers.

3D histology has the potential to transcend these limitations. However, it requires transparency of thick tissue blocks because the opacity inherent to most biological tissues [resulting from light scattering by complex internal structures such as lipids with different refractive indices (RIs) and light absorption by pigments] impedes microscopic examination (*5*). To overcome these physical limitations, lipids are removed, and RIs are matched to the immersion medium. While various tissue clearing methods were invented and have evolved in recent years, human tissue clearing presents particular challenges (*6*).

Because the dimensions of human organs exceed those of common model organisms, human tissue samples can be large. Typical FFPE human tissue blocks (∼2.5 cm by ∼2 cm by up to 1 cm) exceed the size of a mouse brain. Moreover, tissues rich in lipids and collagen such as brain white matter and liver are difficult to clear and stain effectively (*7*–*9*). Also, tissue fibrosis, which increases physiologically with age, makes sample preparation from aged adult patients particularly challenging (*10*, *11*). Thus, processing human samples for 3D histology must achieve effective clearing without compromising tissue integrity and enable complete antibody diffusion without major staining gradients toward the center of the tissue specimen. Both issues are particularly challenging in archival FFPE tissues due to aldehyde cross-linking and epitope masking through formalin fixation, dehydration, and paraffin substitution (*12*). Last, immunofluorescent staining of human samples entails additional issues, including autofluorescence from pigments such as lipofuscin, melanin, or coagulated blood, which can disturb the desired staining signals (*6*).

For these reasons, current human tissue clearing methods with successful immunolabeling are limited to either small (<2-mm-thick) FFPE samples or freshly processed samples from few organs, primarily from the brain (Table 1, table S1) (*13*–*28*). A robust 3D histology pipeline for effective clearing and immunolabeling of large, long-term–stored FFPE human tissue specimens that is applicable across various organs and coupled with a validated set of antibodies remains a major unmet need.

**Fig. 1.**
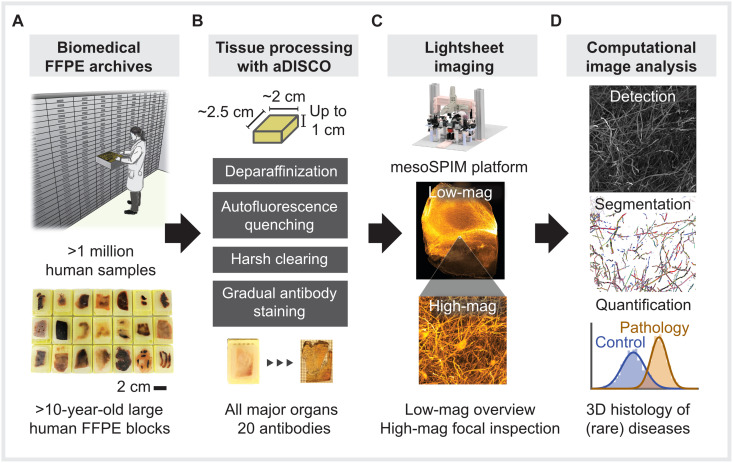
3D histology workflow for entire archival human FFPE tissue blocks using aDISCO. (**A**) Biomedical FFPE archives contain more than a million human samples from all major organs and various pathological backgrounds, often stored for decades. (**B**) aDISCO is designed for processing large human FFPE tissue blocks, and was tested on all major organs and with 20 antibodies. (**C**) Light-sheet imaging with the mesoSPIM platform enables low-magnification (mag) overview and high-magnification focal inspection of cleared and immunolabeled human tissue blocks at (sub-)cellular resolution. (**D**) aDISCO enables quantitative 3D histology of healthy human tissues and (rare) pathologies from FFPE samples using deep learning–based detection and segmentation.

Here, we introduce “archival 3D Imaging of Solvent-Cleared Organs” (aDISCO), a versatile and robust clearing method designed to achieve effective clearing and antibody staining in whole-mount FFPE human tissue blocks from diagnostic archives, thereby making vast collections of human tissue samples available for deeper investigation (Fig. 1) (*29*). We demonstrate the suitability of aDISCO across different human organs and its compatibility with a broad set of antibodies, obtaining whole-mount 3D images at various magnifications using light-sheet fluorescence microscopy [mesoscale selective plane illumination microscopy (mesoSPIM) versions (V) 5 and V6] (*30*, *31*). In a use-case study based on a relatively rare pathological condition [focal cortical dysplasia (FCD)], we demonstrate that 3D histology with aDISCO generates high-quality data that can be processed with automated segmentation and object detection methods based on deep learning. Our results showcase the potential of quantitative 3D histology with aDISCO to detect subtle and focal pathologies in large archival human tissue specimens.

## RESULTS

### Development of aDISCO

**Fig. 2.**
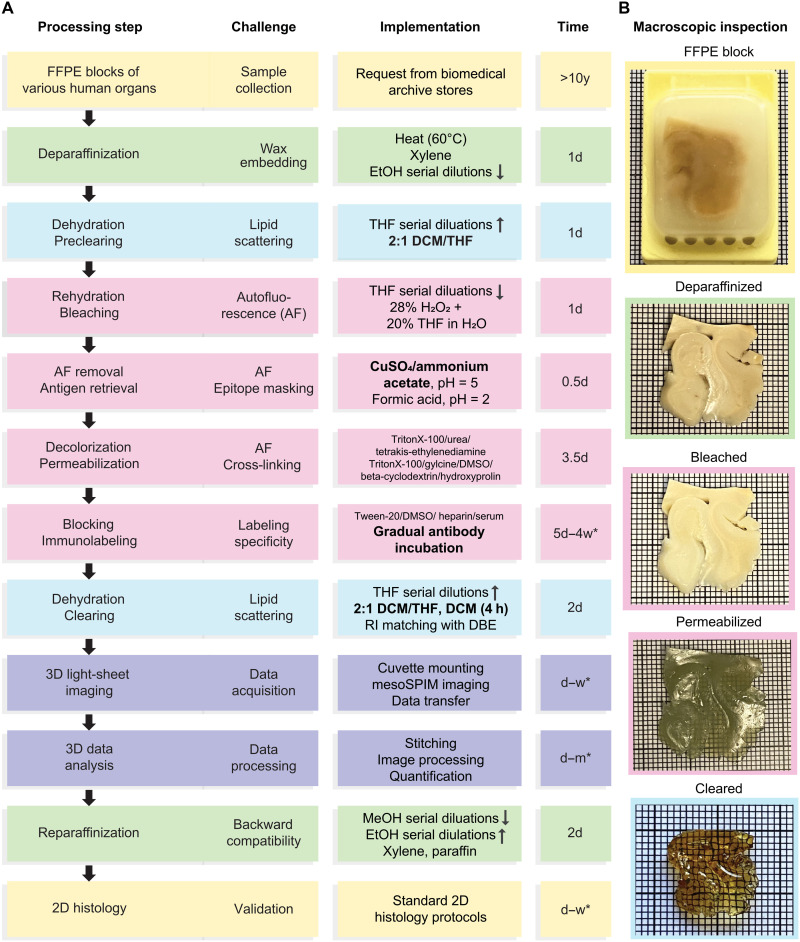
Overview of the aDISCO protocol for clearing and immunolabeling of archival human tissue blocks. (**A**) The aDISCO pipeline (first column), with the associated challenges of processing large archival human tissue blocks (second column), the implementations to overcome these challenges (third column), and the typical times required for the steps (fourth column). FFPE human tissue blocks from biomedical archive stores are processed using harsh deparaffinization, preclearing, and clearing conditions with a mixture of DCM and THF to reach optical transparency. High concentrations of hydrogen peroxide (H_2_O_2_) and cupric sulfate (CuSO_4_) in ammonium acetate remove autofluorescence (AF). Antigen (Ag) retrieval with formic acid helps unmask epitopes after overfixation. Together with preclearing, decolorization, and intense permeabilization, gradual antibody incubation leads to effective antibody diffusion throughout the large and dense tissue blocks. After 3D light-sheet imaging (mesoSPIM) and 3D data analysis, cleared samples can be reparaffinized and reused for conventional 2D histology. DBE, dibenzylether; DMSO, dimethyl sulfoxide; EtOH, ethanol; H_2_O, water; RI, refractive index; h, hours; d, days; m, months; w, weeks; y, years. (**B**) Quality control by macroscopic inspection of an archival human brain sample after serial processing steps as described. Clearing results in about 30% tissue shrinkage. Small squares, 1 mm.

We developed a clearing protocol that enables the processing of long-term-stored (samples used in this study stored for 10 to 15 years) standard FFPE tissue blocks from various human organs, which are customarily stored in diagnostic archives for surgical pathology, autoptic pathology, and at institutes of human anatomy (Figs. 1 and 2A). The method presented here is based on three main innovations: (i) harsh clearing with a combination of tetrahydrofuran (THF) and dichloromethane (DCM), (ii) strong autofluorescence suppression using acidic cupric sulfate in ammonium acetate, and (iii) gradual immunolabeling with increasing antibody concentrations.

Standard FFPE human tissue blocks are relatively large, with the exact sizes depending on sampling and prior cutting. In addition, various tissues including skin, liver, and white matter–rich regions of the brain are very dense (*8*, *9*, *32*). Alcohols used in prior organic solvent–based human tissue clearing protocols (*13*–*15*, *17*) were insufficient to achieve optical transparency in entire archival human tissue blocks, particularly from lipid- and collagen-rich organs (fig. S1A). To overcome the high tissue density and extensive cross-linking in large FFPE human tissues, we developed a harsher clearing strategy based on the combined use of the polar and organic solvents THF and DCM (Fig. 2A and fig. S1A). These chemicals are used repeatedly to achieve stepwise delipidation. In addition, preclearing removes the barriers to molecular flux in dense tissues (*5*), which facilitates antibody penetration for immunolabeling.

Previous clearing protocols used a single antibody concentration, which was often applied at high concentration to reach bright staining signal (*13*, *14*, *17*). However, we observed that in large samples, this procedure typically results in a bright rim and insufficient staining inside the tissue, with a visible signal gradient or even a dark center (fig. S1B). To prevent accumulation of antibodies at the tissue rim, we used an approach starting with low antibody concentration, which we gradually increased by adding more and more antibodies to the staining solution (Fig. 2A and fig. S1B). In addition, we introduced a series of consecutive permeabilization steps before and during the immunolabeling procedure by combining various reagents which have been previously shown to facilitate antibody diffusion (Fig. 2A) (*10*, *33*–*35*). In particular, pretreatment with a permeabilization solution containing tetrakis-ethylenediamine, urea, and Triton X-100 (*33*) before gradual immunolabeling substantially enhanced antibody diffusion and reduced staining time (fig. S1B).

Furthermore, epitopes are often masked in FFPE tissue by overfixation and paraffin embedding, preventing the antibodies from binding to their respective antigens (*12*). The aDISCO protocol already achieved epitope demasking for most tested antibodies through the combined effects of deparaffinization, preclearing, and permeabilization. For antibodies that yielded low signal-to-noise ratio (SNR), we evaluated antigen retrieval methods established in 2D histology and adapted them for use in large tissue blocks. We found that formic acid treatment substantially improved SNR (fig. S1C). In contrast, trypsin treatment only slightly enhanced SNR, treatment with hydrochloric acid or proteinase K adversely affected antigen morphology, and heat-induced antigen retrieval in either sodium citrate buffer (pH = 6) or tris-EDTA buffer (pH = 9) destroyed tissue integrity.

Traditional bleaching agents such as hydrogen peroxide, frequently used in other clearing protocols (*10*, *15*), are often insufficient to suppress the strong autofluorescence from coagulated blood, lipofuscin, melanin, and other pigments (*6*) inherent to human archival tissues (fig. S2A). This persistent autofluorescence is problematic for automated segmentation during 3D data analysis, as brightly autofluorescent structures may be falsely interpreted as positive staining signals. To overcome this limitation, aDISCO incorporates an autofluorescence suppression step using acidic cupric sulfate in ammonium acetate, whose effectiveness has been demonstrated on 2D sections already in the 1990s (*36*). Crucially, we found that its application after immunolabeling suppressed the desired staining signal (fig. S2B). Therefore, we applied it before initiating the staining procedure (Fig. 2A) to preserve the specific staining signal while removing background autofluorescence (fig. S2B).

The combination of these innovations enabled reliable clearing and immunolabeling of entire FFPE tissue blocks from various human organs and was compatible with a substantial set of commonly used antibodies to characterize these organs. An overview of the antibodies tested in different human organs is provided in table S2.

### Validation of aDISCO-processed archival human tissue blocks

Validation of 3D findings by 2D histology requires clearing of archival tissue to be compatible with subsequent reembedding in paraffin, conventional histological processing, and staining (Fig. 2A). We developed a reparaffinization protocol which gently restores the original FFPE tissue block integrity and results in 2D sections with similar staining quality as the original noncleared FFPE tissue sections (fig. S2C).

Tissue clearing procedures are laborious and can take several weeks depending on sample size. It is therefore helpful to assess their performance at intermediate stages. We performed such quality testing on archival human brain samples during aDISCO processing (Fig. 2B). After deparaffinization, archival human tissue was of brownish color, which became whitish after successful bleaching and greenish after cupric-sulfate treatment. After strong permeabilization and decolorization, less compact tissues such as the cortical gray matter of the brain already started becoming transparent, whereas the white matter remained opaque. Final clearing and RI matching rendered the entire sample fully transparent.

aDISCO clearing induced tissue shrinkage of about 30% compared to the rehydrated state following deparaffinization in most tested human organs (table S3). Shrinkage was highest in the skin, which contains abundant subcutaneous fat, and lowest in tissues with highly ordered, longitudinal fiber architecture. While shrinkage was nearly isotropic in most organs, peripheral nerve, liver, and lung exhibited moderate anisotropic shrinkage, likely reflecting their heterogeneous and compartmentalized sample architecture.

### High-quality clearing and immunolabeling throughout large archival human tissue blocks

**Fig. 3.**
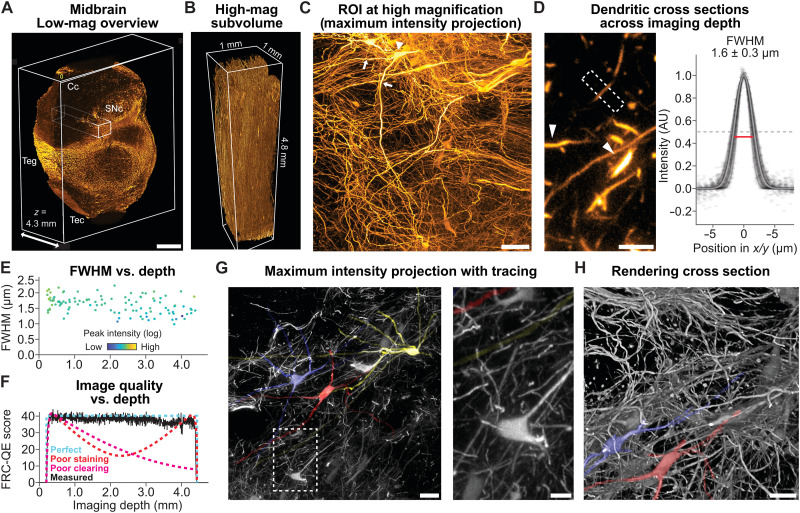
Large-scale screening and high-resolution analysis of archival human midbrain. (**A**) 3D rendering of archival human midbrain, stained for dopaminergic neurons (anti-TH) and imaged at low optical magnification (1.25x). The rendering shows the crus cerebri (Cc), tegmentum (Teg), tectum (Tec), and substantia nigra pars compacta (SNc). Scale bar, 2 mm. (**B**) Subvolume of the SNc at high magnification (12.6x). (**C**) Maximum intensity projection of the high-magnification subvolume highlights large dopaminergic neurons with somata (arrowhead) and neurites (arrows), branching into a dense fiber network. Scale bar, 50 μm. (**D**) Left: Thin dendrites (dashed white rectangle) are selected for cross-sectional profiling as a readout of lateral resolution across sample depth. Bright and thick dendrites (arrowheads) are avoided for analysis. Right: Dendritic cross-sectional intensity profiles with Gaussian fits in arbitrary units (AU; *N* = 108 across all depths). The full width at half maximum (FWHM; median ± SD) was obtained from Gaussian fits to individual profiles. (**E**) The lateral FWHM shows no depth-dependent increase. Peak intensities of dendritic profiles are color-coded (logarithmically spaced in AU). (**F**) Fourier ring correlation quality estimate (FRC-QE) across imaging depth. Ideally, FRC-QE is constant across imaging depth (light blue). For poorly stained samples, FRC-QE would be high at the sample edges and low in the center (red). For poorly cleared samples, FRC-QE would decrease with imaging depth (magenta). The measured FRC-QE is stable across imaging depth (black). (**G**) Maximum intensity projections across 30 μm with tracing of the main neuronal processes in the sample center, shown here for three example neurons with distinct color coding. The inset highlights small neuronal processes. (**H**) 3D rendering of traced neuronal processes. See movie S2.

To assess the performance of the aDISCO protocol, we processed an archival human midbrain sample with a high content of lipid-rich white matter, stored as FFPE for >10 years. We stained the sample with anti–tyrosine hydroxylase (TH) antibodies that visualize dopaminergic neurons (Fig. 3A and movie S1). In a 3D rendering of the whole-mount imaged sample using light-sheet microscopy, the architecture of the human midbrain, including the substantia nigra, can be identified (Fig. 3A). These results were reproducible across independent samples (fig. S3A).

Region-of-interest (ROI) light-sheet imaging of the substantia nigra with higher magnification highlights branching neurites, including fine dendrites (Fig. 3, B and C).

Optical inhomogeneities lead to light scattering and result in a reduction of the effective imaging resolution. The nominal lateral resolution of our imaging method is 1.7 μm. To demonstrate effective clearing and immunolabeling throughout sample depth, small and ideally subdiffractive objects can be inspected. To this end, we analyzed the cross-sectional profiles of small neuronal dendrites across depth (Fig. 3D), with no obvious increase of dendritic cross-sectional width with depth, indicating preserved lateral resolution throughout the full thickness of the cleared sample (Fig. 3E). The variability of measured dendritic width can be explained by varying peak intensities of the inspected dendrites, indicating that thicker dendrites slightly broadened the profiles due to their thickness or due to saturation effects. A complementary analysis based on Fourier ring correlation analysis (*37*) confirmed that image quality was relatively stable across imaging depth (Fig. 3F). To demonstrate that this high image quality enables the analysis of subcellular structures, we used the neuronal tracing framework webKnossos (*38*) to track the main processes of selected neurons within the center of the high-magnification subvolume (Fig. 3, G and H, and movie S2).

Together, these analyses indicate that aDISCO enables imaging at high, even subcellular resolution across the entire depth of lipid-rich archival human tissue blocks.

### 3D histology of different human CNS regions

**Fig. 4.**
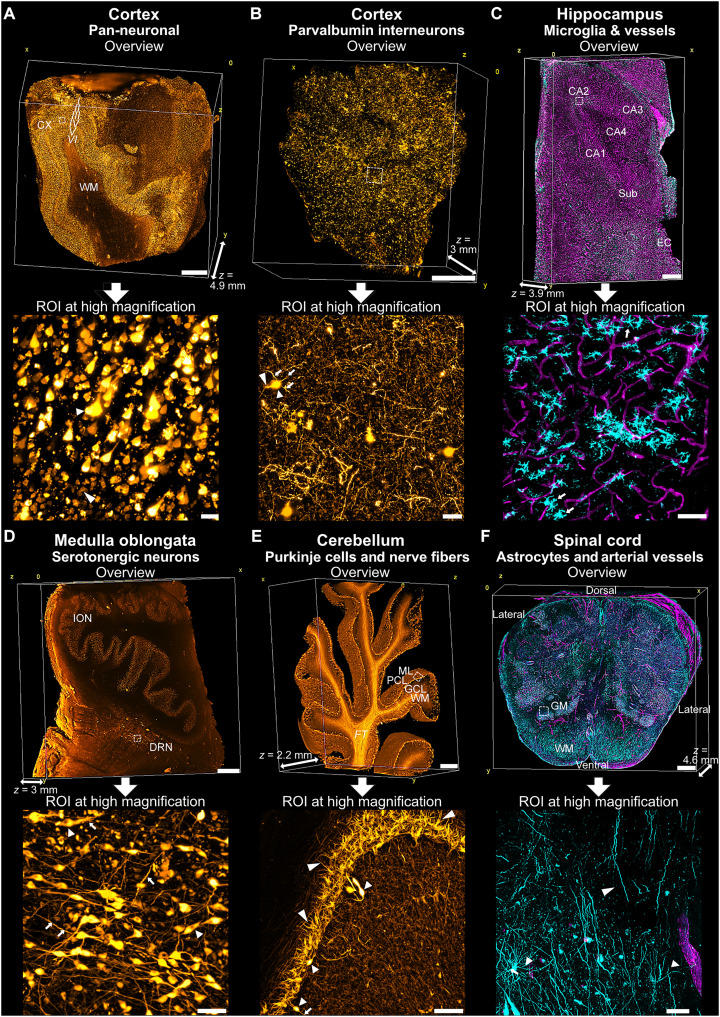
3D histology of different human CNS regions. Each panel presents a low-magnification 3D rendering of a whole-mount archival human sample (top; 0.8× to 2×) and a high-magnification maximum intensity projection of a region-of-interest (ROI; bottom; 12.6×; dashed boxes in rendering). (**A**) Brain cortex (CX) sample stained for neurons (anti-NeuN), revealing cortical layers I to VI and white matter (WM). Scale bar, 2 mm. The ROI highlights granular cells (large arrowhead) and pyramidal cells (small arrowhead) in layer III. Scale bar, 50 μm. (**B**) Parvalbumin-positive interneurons in brain cortex. Scale bar, 1 mm. The ROI shows neuronal somata (small arrowhead), axons (large arrowhead), and dendrites (arrows). Scale bar, 100 μm. (**C**) Microglia (cyan; anti-IBA1) and blood vessels (magenta; anti-podocalyxin) in the hippocampus across anatomical subregions [cornu ammonis (CA)4, CA3, CA2, CA1, subiculum (Sub), and entorhinal cortex (EC)]. Scale bar, 1 mm. The ROI reveals microglia processes (arrows) close to blood vessels. Scale bar, 50 μm. (**D**) Serotonergic neurons (anti-TPH2) in the medulla oblongata, with the inferior olivary nucleus (ION) and the dorsal raphe nucleus (DRN). Scale bar, 1 mm. The ROI displays serotonergic neurons with nuclei (arrowheads) and ramified neurites (arrows). Scale bar, 50 μm. (**E**) Cerebellum (anti-neurofilament) with WM fiber tracts (FT) and cerebellar layers: molecular layer (ML), Purkinje cell layer (PCL), and granular cell layer (GCL). Scale bar, 1 mm. The ROI displays Purkinje cell somata (small arrowheads) with dendrites (large arrowheads) and axons (arrow). Scale bar, 50 μm. (**F**) Astrocytes (cyan; anti-GFAP) and arterial vessels (magenta; anti-SMA) in the spinal cord. Gray matter (GM) can be distinguished from white matter (WM) based on distinct astrocytic staining patterns. Scale bar, 1 mm. The ROI displays stellate astrocytes (arrow) and glial processes (large arrowhead) besides arterial vessels (small arrowheads). Scale bar, 50 μm.

Next, we tested the applicability of aDISCO across a broad spectrum of human archival central nervous system (CNS) samples (Fig. 4 and movies S3 and S4). We used various antibody stains and light-sheet microscopy to visualize neuronal nuclei (NeuN)-positive neurons or specifically parvalbumin-positive interneurons in the cortex; ionized calcium-binding adapter molecule (IBA1)-positive microglia and podocalyxin-positive blood vessels in the hippocampus; tryptophane hydroxylase 2 (TPH2)–positive serotonergic neurons in the brain stem; neurofilament-positive neurons, including Purkinje cells, and nerve fibers in the cerebellum; and glial fibrillary acidic protein (GFAP)–positive astrocytes and smooth muscle actin (SMA)–positive arterial vessels in the spinal cord. The reproducibility of selected stains in independent samples is shown in fig. S3 (B and C).

To assess the clearing and immunolabeling quality in the sample center, we performed orthogonal reslicing of an anti-NeuN–labeled human cortex sample that exhibits an evenly distributed staining pattern across the entire cortex and is therefore suitable for this type of analysis (fig. S4A). In all three image projections, staining signals in the cortex were preserved throughout the reslices. In addition, both cortex and lipid-rich white matter did not exhibit obvious signs of blurring, indicating effective clearing of the entire sample. Next, we calculated the mean fluorescence intensity (MFI) projection along the *z* direction of the entire sample. In the *x* and *y* directions, MFI was similar between superficial and deep areas of similar composition, with inhomogeneities reflecting region-specific cellular distributions rather than staining gradients (fig. S4B; see fig. S5 for MFI projections of the other CNS samples). In addition, we computed the *z*-profile of the MFI averaged across *x* and *y*. The *z*-profile remained stable throughout the entire range in the vertical (*z*) direction, even in the center of the sample (fig. S4C). In general, imaging depth was not limited by antibody penetration or clearing quality but by the size of the respective tissue blocks (Figs. 3 and 4), which was 8.7 ± 2.8 mm (mean ± SD across *N* = 7 CNS samples) × 7.5 ± 1.9 mm × 3.7 ± 1.0 mm after clearing-induced tissue shrinkage. Accordingly, the maximum imaging depth was 4.9 mm, corresponding to the entire sample thickness of the anti-NeuN–stained archival human cortex sample after clearing and accompanying tissue shrinkage (Fig. 4A).

To further make use of the high optical resolution enabled by aDISCO, we imaged ROIs across all processed CNS samples at higher magnification (insets in Fig. 4). This allowed us to identify individual somata and neurites of several neuronal cell types (Fig. 4, A, B, D, and E), the interactions of microglia feet with finely branched blood vessels (Fig. 4C), and the processes of stellate astrocytes in the spinal cord (Fig. 4F).

In summary, we found that aDISCO achieved effective clearing and immunolabeling of large archival human samples across the entire CNS spectrum while enabling subcellular resolution of fine structures even deep within each tissue block. Because the staining quality did not degrade after up to 15 years of storage (anti-NeuN–labeled cortex sample), aDISCO seems suitable for the investigation of historical samples.

### 3D histology of human organs beyond the CNS

**Fig. 5.**
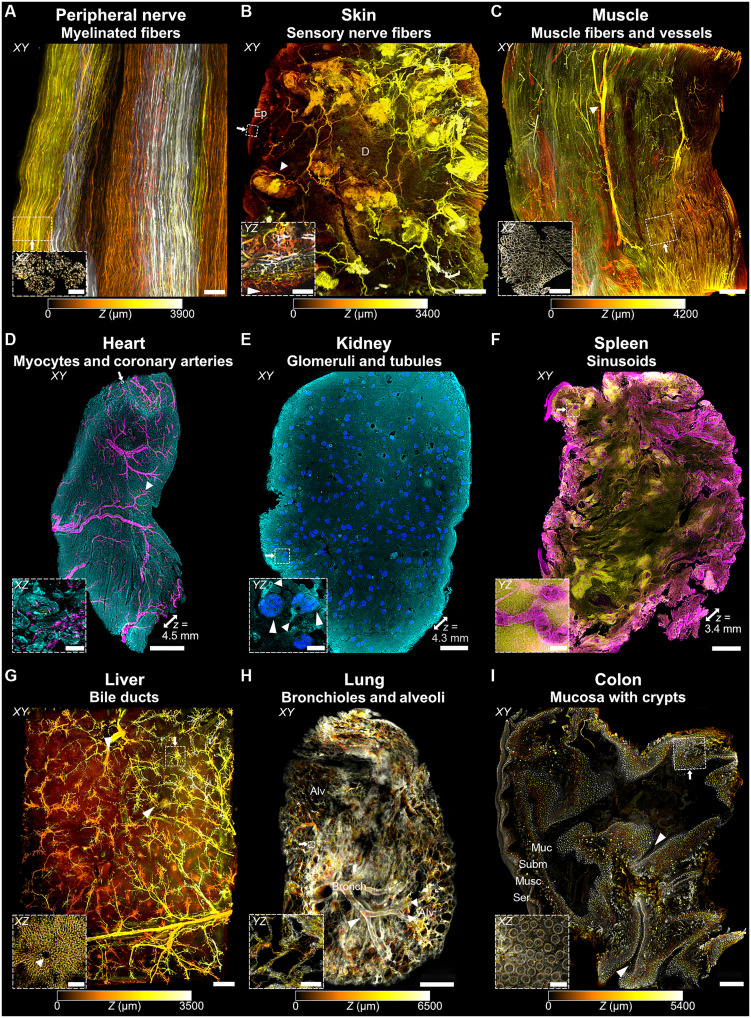
3D histology of human organs beyond the CNS. Overview panels (0.63× to 12.6×) with depth color encoding (A to C and G to I) or as maximum intensity projections (D to F). Orthogonal ROI views (4× to 20×) are indicated by dashed boxes, and viewport direction is illustrated by arrows. All numerical values indicate scale bar lengths. (**A**) Myelinated fibers (anti-MBP) in peripheral nerve, displaying a cotton wool-like pattern (500 μm), and nerve fascicles (inset; 100 μm). (**B**) Sensory nerve fibers (anti-PGP9.5) in the skin, branching in epidermis (Ep) and dermis (D) and forming Pacinian corpuscles (arrowhead in overview; 1 mm). Inset: Innervation of epidermis by nerve terminals (arrowhead; 100 μm). (**C**) Muscle fibers (anti-laminin) in skeletal muscle, with vascularization (arrowhead in overview; 1 mm) and muscle fascicles (inset; 200 μm). (**D**) Myocytes (cyan; anti-N-cadherin) and coronary arteries (magenta; anti-SMA) in the heart, with coronary artery branches within the myocardium (arrowhead in overview; 2 mm) and arterioles between myocytes (inset; 30 μm). (**E**) Glomeruli (anti-Nephrin) and tubules (anti-E-cadherin) in the kidney (overview; 1 mm), highlighting Bowman capsules (large arrowheads in inset; 150 μm) and outgoing proximal tubules (small arrowheads). (**F**) Sinusoids (anti-SMA) and lymphatic tissue (anti-CD45) in the spleen. Overview: 1 mm. Inset: 100 μm. (**G**) Bile ducts (anti-CK19) branching in the liver (arrowheads in overview; 1 mm) and a hepatic lobule with central vein surrounded by bile ducts (arrowhead in inset; 200 μm). (**H**) Airways in the lung (anti-CK7), with bronchioles (Bronch) branching (large arrowhead in overview; 2 mm) and segueing (small arrowheads) into alveoli (Alv), and detailed view of alveoli (inset; 100 μm). (**I**) Colon sample (anti-Collagen-4), showing mucosal crypts (arrowheads; 1 mm) within the colon wall composed of mucosa (Muc), submucosa (Subm), muscularis (Musc), and serosa (Ser), and individual crypts across inner mucosa to outer serosa (double-headed arrow in inset; 300 μm).

In principle, aDISCO should be applicable to human organs other than the CNS, but adaptations may be needed because of differences in tissue composition. We therefore processed whole-mount FFPE human tissue blocks from disparate organs, stained them with a panel of antibodies, and imaged them by light-sheet microscopy (Fig. 5 and movies S5 and S6). With aDISCO, we visualized myelinated fibers in peripheral nerve, sensory nerve fibers in the skin, skeletal muscle fibers together with their vascularization, cardiac myocytes and coronary arteries in the heart, glomeruli and tubules in the kidney, sinusoids in the spleen, bile ducts in the liver, bronchioles and alveoli in the lung, and the mucosa with crypts in the colon. The reproducibility of selected stains in independent samples is shown in fig. S6.

These examples demonstrate that aDISCO achieved effective clearing and immunolabeling of cellular structures across a broad variety of human organs. This finding was supported by MFI projections, showing robust staining signals in each tissue block (fig. S7). This was also true for very dense tissues, such as liver or skin. Again, imaging depth was not limited by antibody penetration or clearing quality but by the size of the respective tissue blocks, which was 10.7 ± 4.7 mm (mean ± SD across *N* = 9 samples) × 7.6 ± 3.2 mm × 4.3 ± 1.5 mm after clearing-induced tissue shrinkage. Accordingly, the maximum imaging depth was 6.5 mm, corresponding to the entire sample thickness of the anti-CK7–stained archival human lung sample after clearing and accompanying tissue shrinkage (Fig. 5H).

The clearing and staining quality enabled the resolution of thin structures (insets in Fig. 5), including the branches of sensory nerve terminals in the epidermis of the skin (inset in Fig. 5B and movie S5) and the Bowman capsules and outgoing proximal tubules of glomeruli in the kidney (inset in Fig. 5E and movie S6).

In conclusion, our findings highlight the versatility of aDISCO for 3D histology of whole-mount FFPE human tissue blocks across multiple organs and its compatibility with a broad range of antibodies for detailed organ characterization.

### 3D cortical profiling of neuronal density in archival human brain

Because large cleared samples can be imaged volumetrically using 3D microscopy, aDISCO may enable the 3D quantitative morphometry of tissues such as human cortex with analytical precision that is unattainable by conventional histology. The human cortex exhibits a characteristic architecture consisting of six neuronal layers (*39*) and is essential for performing complex neuronal computations, which are the basis of cognition and behavior. The precise quantification of cortical neurons and their distribution across distinct layers may help advance the understanding of the relationships between functional integrity and cellular organization in the human cortex. This knowledge may be relevant to many fields of neuroscience, from refining models of neuronal computation to elucidating structural alterations during development, aging, and in pathological states.

**Fig. 6.**
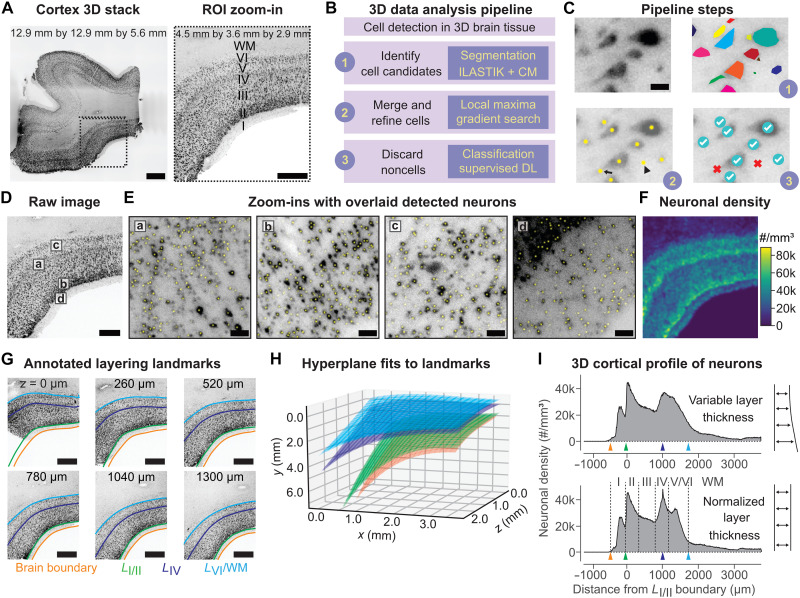
Neuronal density profiling in archival human brain cortex. (**A**) Left: Stitched 3D stack of whole-mount archival human cortex specimen (4x magnification), stained for neurons (anti-NeuN). Scale bar: 2 mm. Right: Zoom into an ROI, highlighting cortical layers I to VI and the white matter (WM). Scale bar, 1 mm. (**B**) 3D data analysis workflow for neuronal cell detection in three steps. CM, ClearMap; DL, deep learning. (**C**) Illustration of the pipeline steps explained in (B). Upper left: Raw fluorescence image. Scale bar, 20 μm. Top right: Segmented neuronal cell candidates, visualized by different colors. Bottom left: Detection of cortical neurons based on local maxima (yellow dots) of segmented neuronal cell candidates. Note false-positive detection of axons separated from somata (arrow) and of noise (arrowhead). Bottom right: Falsely detected cell candidates are discarded (red crosses) via supervised DL, while correctly detected neurons are kept (cyan check marks). (**D**) Raw fluorescence image of cortical layers. Scale bar, 1 mm. (**E**) Zoom-in micrographs of regions numbered in (D), with overlaid detected neurons (yellow dots). Scale bars, 50 μm. (**F**) Spatial mapping of neuronal density. *#*, neuron count in thousands (k). (**G**) 3D annotation of visually identified layering landmarks for defining distinct layers (*L*), indicated by the different colors and exemplarily shown along the *z* axis. Scale bars, 1 mm. (**H**) Generation of polynomial hyperplanes fitted to the respective layering landmarks. (**I**) Neuronal density in thousands per mm^3^ across all cortical layers, defined by the landmark hyperplanes in (H) as indicated by the colored arrows and plotted according to the distance from the boundary between layers I and II (*L*_I/II_). 3D cortical profile before (upper histogram) and after normalization to variable layer thickness (lower histogram).

We therefore performed 3D histology with anti-NeuN immunolabeling in large archival FFPE human cortex specimens for detailed 3D profiling of neuronal density across distinct cortical layers (Fig. 6).

We devised a 3D data analysis pipeline for efficient and accurate neuron counting in large datasets (Fig. 6, B and C). We used ILASTIK (*40*) and ClearMap (*41*) for segmentation and detection of neuronal cell candidates, which were subsequently refined by merging falsely separated parts of neurons. Neuronal candidates were then classified as cells versus noncells using a supervised deep convolutional neural network (DCNN) that used three orthogonal virtual slices of the local environment of each candidate to predict its class (cross-validated accuracy 92.2%; fig. S8).

This approach enabled robust detection of neurons and subsequent determination of neuronal density in human cortex (Fig. 6, D to F, and movie S7). To define cortical layers, we used visual guidance to annotate landmarks that indicate specific layers or boundaries between layers and fitted them to polynomial hyperplanes. This approach enabled precise determination of neuronal densities across distinct cortical layers (Fig. 6, G and H). To compensate for variable cortical thickness within a 3D sample, we normalized layer thickness by the average distance between landmarks. As a result, we obtained a layer-dependent neuronal density profile that can distinguish distinct cortical layers, except layers V and VI where the transition is also morphologically less well-defined (Fig. 6I).

In summary, our analyses show that large archival human samples processed via aDISCO provide clearing and antibody staining quality that is amenable to segmentation and object detection. Our 3D data analysis pipeline allows for accurate detection of neurons and delineation of neuronal densities across distinct cortical layers in three spatial dimensions. By establishing a reference profile of neuronal cell distribution in normal human cortex, we provide a basis for assessing cortical abnormalities under pathological conditions.

### 3D histological investigation of FCD

To test a use-case for these tools, we investigated the cortical architecture in FCD, a neurodevelopmental malformation disorder causing pharmaco-resistant epilepsy in children and young adults (*42*). Its prevalence within the general population is <1% (*43*) but ranges between 5% and 25% among patients suffering from focal epilepsy (*44*). FCD is characterized by distortions in cortical layer architecture and is classified into three different types depending on the histopathological phenotype (*45*, *46*). Type I is characterized by isolated abnormal radial and/or tangential cortical lamination. Type II is the most prevalent form and characterized by dysmorphic neurons (subtype IIa) or additionally “balloon cells” (subtype IIb), which represent nonfunctional neural progenitor cells with enlarged cell bodies. Type III is associated with other pathologies such as hippocampal sclerosis, neoplasia, or vascular malformations. Ectopic neurons within the white matter can be found in all FCD types.

The diagnostic assessment of FCD in thin 2D histological sections suffers from several limitations. First, the inherent focal nature of FCD makes it often difficult to identify pathological foci in large surgical specimens obtained from complete epileptic zone excision (*47*–*49*). This requires exhaustive and time-consuming serial sectioning. Second, suboptimal brain tissue orientation during FFPE block embedding and sectioning may impede accurate evaluation of cortical layering, which is crucial for FCD diagnosis. Last, FCD can manifest multifocally (*42*), rendering single thin sections inadequate for portraying the full pathological spectrum. The latter consideration is pivotal for estimating disease severity and devising appropriate follow-up interventions. Therefore, we were curious to assess whether 3D histology could help improve FCD diagnostics in difficult cases and accurately assess the extent of pathology in the entire surgical specimen. The compatibility of aDISCO with FFPE specimens from diagnostic archives allowed us to retrieve adequate numbers of historical specimens and benchmark 3D histology against traditional analyses.

We selected seven FCD cases confirmed by 2D histology from the diagnostic archive of the University Hospital of Zurich (table S4). All patients had undergone surgery due to refractory epilepsy. Only in three cases could the pathology be assessed throughout all cortical layers using conventional histology, due to the limitations of 2D techniques. For control, we selected seven autopsy samples of age-matched patients with normal histology in the respective cerebral lobes from the diagnostic archive of the University Hospital of Zurich.

**Fig. 7.**
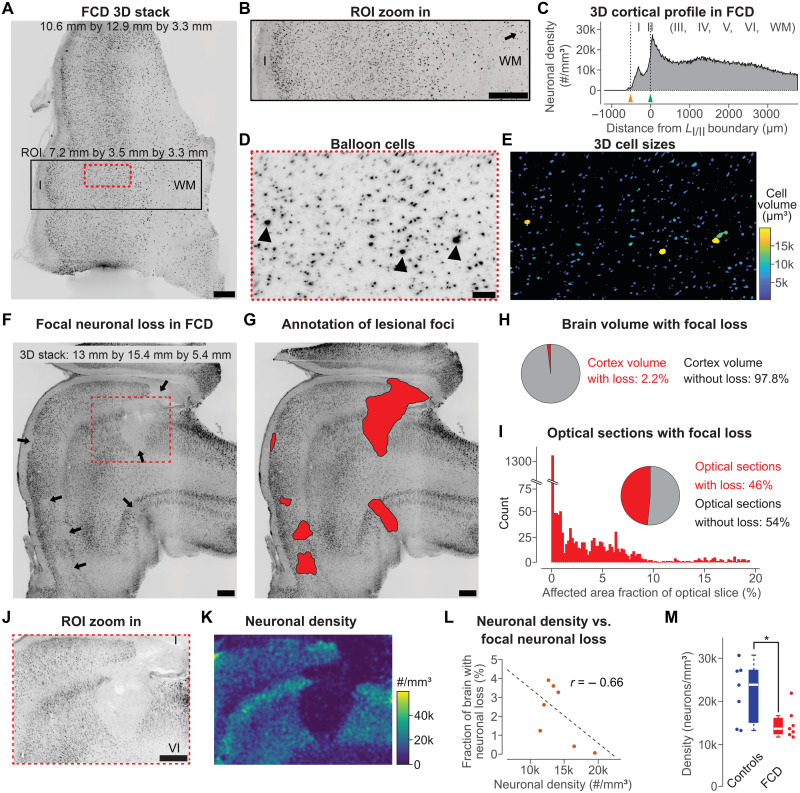
3D histological examination of FCD. (**A**) Stitched 3D stack of archival human FCD cortex (4× magnification), stained for neurons (anti-NeuN). Scale bar, 1 mm. (**B**) Zoom-in of the black-lined box, highlighting distortion of cortical layer architecture and ectopic neurons in the white matter (WM; arrow). Scale bar, 1 mm. (**C**) Neuronal density profiling in FCD, displayed as number (*#*) of neurons per mm^3^ across all cortical layers, measured as the distance from the *L*_I/II_ boundary. (**D**) Zoom-in of the red-dashed box, highlighting large, rounded balloon cells (arrowheads). Scale bar, 200 μm. (**E**) Automated detection of balloon cells based on their large cell size [color-coded cell volume in thousands (k)]. (**F**) Stitched 3D stack of archival human FCD cortex (4× magnification), stained for neurons (anti-NeuN), showing multiple foci of neuronal loss (arrows). Scale bar, 1 mm. (**G**) 3D annotation of lesional foci. Scale bar, 1 mm. (**H**) Quantification of lesional foci in entire brain tissue blocks. (**I**) Quantification of lesional foci in optical sections. The histogram displays the count of optical sections in relation to the affected area per optical slice. (**J**) Zoom-in of the red-dashed box, highlighting focal neuronal loss across cortical layers I to VI. Scale bar, 500 μm. (**K**) Quantification of overall neuronal density from the zoom-in (J), displayed as number (*#*) of neurons per mm^3^ in a heatmap. Cell number range in thousands (k). (**L**) Correlation of focal neuronal loss in percentage (%) and overall neuronal density displayed as number (*#*) of neurons in thousands (k) per mm^3^ (*r* = −0.66). (**M**) Comparison of overall neuronal density quantification between patients with FCD (red; *N* = 7) and controls (blue; *N* = 7), displayed as number of neurons in thousands per mm^3^. Statistical analysis was performed by permutation test. *P = 0.032.

We stained the neurons with anti-NeuN antibodies to assess neuronal density across all cortical layers for 3D cortical profiling. Qualitative inspection of 3D stacks revealed focal distortion of the typical cortical layer architecture (see Fig. 6A), with only the molecular layer (I), the external granular layer (II), and the beginning of the white matter (WM) distinguishable, but not the layers in between (Fig. 7, A and B). Furthermore, we observed ectopic neurons in the white matter. In contrast to the reference profile of normal cortex (see Fig. 6I), the cortical neuronal density profile in FCD exhibited only two peaks for layers I and II, while the other layers merged into a single hill that flattened slowly toward the white matter (Fig. 7C). This quantitative analysis demonstrates that 3D cortical profiling of neuronal density reflects the distortion of cortical layer architecture in FCD.

To investigate an additional hallmark of FCD, we quantified the cell volume. This analysis correctly detected the characteristic large, rounded balloon cells of FCD type IIb (Fig. 7, D and E). Furthermore, during visual inspection of 3D stacks from patients with FCD, we noticed multiple foci of neuronal loss, substantially varying in size and shape within and between patients (Fig. 7F). Based on the clinical history of FCD patients, these lesions were not caused by electrode implantations for two reasons: First, some patients who exhibited lesional foci had not undergone any electrode implantations. Second, for those who had been implanted with electrodes, the lesional foci did not match the electrode pattern in size and shape.

To analyze the extent of focal neuronal loss, we annotated all lesions in the entire 3D stacks (Fig. 7G). The quantitative volumetric analysis of neuronal loss foci demonstrated that these lesions affected only 2.2% of the cortical volume across FCD samples (Fig. 7H and table S5). When we analyzed the 3D stacks as optical *xy* sections along the *z* axis, foci of neuronal loss were detected in less than half of optical sections (Fig. 7I). Most lesional foci were small, and the maximally affected area per optical slice was 20%. Hence, the chance of missing these foci in thin sections, as in conventional 2D diagnostics, is high.

Last, we quantified the overall neuronal density in brain volumes, including the lesional foci and the surrounding inconspicuous brain tissue in FCD (Fig. 7, J and K). Bivariate analysis showed a strong negative correlation between overall neuronal density and the extent of lesional foci in patients with FCD, indicating that FCD specimens with a higher fraction of brain volume with focal loss also exhibited overall lower neuronal density (Fig. 7L). Comparison between FCD and control groups revealed a significant reduction of neuronal density in patients with FCD (median reduction of 43%; Fig. 7M and tables S5 and S6). Thus, the overall neuronal loss in FCD (43%; Fig. 7M) exceeded the loss caused by the lesional foci alone (2.2% of cortical volume; Fig. 7H) and affected the brain tissue that would have been classified as healthy by conventional 2D histology. Therefore, quantitative 3D histology revealed additional neuronal loss undetectable by visual inspection. In summary, our analyses demonstrate that aDISCO can be used to address specific pathologies by comprehensive and quantitative 3D histology, facilitated by a largely automated 3D data analysis pipeline.

## DISCUSSION

Here, we introduce aDISCO, a tissue clearing method for 3D histology of whole-mount FFPE human tissue blocks, as a complementary diagnostic technique to conventional 2D histology. We demonstrate that aDISCO achieves effective clearing and antibody staining throughout entire archival tissue blocks from different human organs and is compatible with a variety of antibodies, enabling specific immunolabeling of target structures down to the cellular level.

A central feature of aDISCO is the effective clearing and antibody staining of whole-mount FFPE human tissue blocks, whose sizes surpass the state of the art for human FFPE specimens (Table 1) (*14*, *19*, *26*, *28*). Imaging these large samples at cellular resolution with high throughput has only recently become a widely used approach due to the development of light-sheet microscopes with large working-distances (*30*, *31*, *50*). The largest human samples cleared and stained to date were freshly processed with the SHANEL protocol (*17*, *18*). In this organic solvent–based tissue clearing method, the blood vessels of the respective human organs were perfused with chemical reagents to circumvent the diffusion barrier. With this procedure, a maximum imaging depth of 1 cm was reached for antibody-stained tissue. However, this approach is not applicable to archival human FFPE specimens from diagnostic archives, which contain large numbers of human samples from the distant past and thus constitute an untapped potential to study specific and rare diseases. The greatest imaging depth in FFPE tissue so far had been achieved with the organic solvent–based DIPCO protocol (*14*). However, DIPCO was applied to short-term FFPE human biopsies (usually fixed <24 hours) as opposed to long-term FFPE human tissue specimens from autopsy (usually fixed >24 hours up to 3 weeks, depending on organ type and size). In the DIPCO study, a maximum imaging depth of 1.6 mm was reached in human tumor biopsy specimens from six different human organs, characterized with four antibodies. To date, the only published human tissue clearing method performed on long-term FFPE human autopsy tissue was OPTIClear (*19*). With a combination of organic solvents used for deparaffinization and a CLARITY-related clearing approach, this method reached a maximum imaging depth of 546 μm for antibody staining in human brain tissue. In contrast, in our study, we reached a maximum imaging depth of 6.5 mm throughout the entire tissue block, with the sizes of the available samples being the limiting factor for the maximum imaging depth. The archival human FFPE tissue samples in our study had a maximum image size of 18.5 mm by 11.9 mm by 6.5 mm (lung sample) after clearing-induced tissue shrinkage of about 30% (table S3) (*51*), i.e., with an original size on the centimeter scale. The average sample size after clearing was 9.8 ± 4.2 mm × 7.6 ± 2.6 mm × 4.1 ± 1.0 mm^3^ (mean ± SD across 16 samples). Thus, with aDISCO, we have established a tissue clearing method that enables access to whole-mount archival human tissue blocks by being compatible with both large human tissue specimens and samples from long-term FFPE storage (up to 15 years old in our study).

**Table 1.** Comparison of human tissue clearing methods. iDISCO, immunolabeling-enabled dimensional imaging of solvent-cleared organs; DIPCO: diagnosing immunolabeled paraffin-embedded cleared organs; MASH, multiscale architectonic staining of human cortex; SHANEL, small micelle-mediated human organ efficient clearing and labeling; aDISCO, archival 3D imaging of solvent-cleared organs; OPTIClear, optimal clearing; FASTClear, fast clearing; CLARITY, clear lipid-exchanged acrylamide-hybridized rigid imaging/immunostaining/in situ-hybridization-compatible tissue hydrogel; SWITCH, system-wide control of interaction time and kinetics of chemicals; SHIELD, stabilization under harsh conditions via intramolecular epoxide linkages to prevent degradation; HIF, heat-induced ffpe-based tissue clearing; ELAST, elasticizing tissues; CUBIC, clear, unobstructed brain/body imaging cocktails; FFPE, formalin-fixed paraffin-embedded; Ab, antibody; No., number; N/A, not applicable.

	iDISCO (*13*)	DIPCO (*14*)	MASH (*15*, *16*)	SHANEL (*17*, *18*)	aDISCO	OPTIClear (*19*)	FASTClear (*20*, *21*)	CLARITY (*22*, *23*)	SWITCH (*24*) SHIELD (*25*)	HIF (*26*)	ELAST (*27*)	CUBIC (*28*)
FFPE	No	Yes	Yes	No	**Yes**	Yes	No	No	No	Yes	No	Yes
Long-term formalin-fixed	No	No	Yes	No	**Yes**	Yes	No	Yes	Yes	No	Yes	No
Imaging depth	<1 mm	1.6 mm	3 mm (no Ab)	1 cm	**6.5 mm**	546 μm	< 509 μm	2.6 mm	100 μm	900 μm	5 mm	2.1 mm (FFPE no Ab), 400 μm (fresh)
Complete antibody penetration	Yes	Yes	N/A (no Ab)	No	**Yes**	Yes	Yes	No	Yes	Yes	No	N/A (FFPE no Ab), yes (fresh)
No. of human organs	1	6	2	7	**11**	1	3	1	1	1	1	3
No. of primary antibodies	2	4	0	10	**20**	8	3	9	10	3	12	0 (FFPE), 2 (fresh)

A second unique feature of aDISCO is its tested compatibility with 11 different human organs. This broad range of applications surpasses all existing clearing methods to date. Previous human tissue clearing methods were typically performed on one or a few tissue types (Table 1 and table S1). Most studies focused on human brain cortex (*13*, *15*, *17*, *19*, *20*, *22*–*27*), which is relatively straightforward to render transparent. In contrast, reports on dense tissues such as liver or skin, which are more challenging to clear (*9*, *32*), are sparse. In human skin, previous work achieved a maximum imaging depth of 300 μm in immunolabeled tissue, while a depth of 1.4 mm was reached in unstained, autofluorescent tissue (*52*, *53*). Similarly, in human liver, most studies achieved imaging depths of only a few hundred micrometers in antibody-stained tissue (*54*–*57*). Notably, only two studies successfully imaged human liver volumes of 2.5- and 3-mm thickness, respectively (*58*, *59*). However, human tissue clearing of archival FFPE skin or liver tissue had not been previously performed. With aDISCO, we achieved imaging depths of 3.4 mm in immunolabeled archival human skin and 3.5 mm in immunolabeled archival human liver tissue, values that again were only limited by the thickness of the available whole-mount FFPE blocks.

The third key advantage of aDISCO is its compatibility with at least 20 commonly used antibodies for cell typing, surpassing previous human tissue clearing studies. Antibody compatibility has been a substantial challenge in tissue clearing, and prior testing of antibodies with the specific chemicals and conditions used in clearing protocols has therefore been recommended (*10*, *60*). Most antibodies were so far tested for compatibility only in nonarchival human tissue (*17*, *18*, *25*, *27*) (Table 1 and table S1). The study with the largest (3-mm-thick) FFPE human tissue specimens to date used staining with low-molecular weight dyes (*16*), which, compared to antibodies, diffuse more easily into thick tissues but lack the ability to differentiate distinct cell types. aDISCO overcomes these limitations, providing compatibility with diverse antibodies and complete immunolabeling throughout whole-mount archival human tissue blocks.

Last, the ability to reparaffinize the cleared tissue blocks highlights the protocol’s versatility for subsequent conventional 2D histology, which is essential for backward compatibility and validation with the state-of-the-art method.

In a use-case study, we show that aDISCO enables standardized quantitative 3D histology of human samples collected from the diagnostic archive and overcomes key limitations of conventional 2D diagnostics. The characterization of human cortical layers (*61*) has relied primarily on thin 2D sections (4 to 5 μm) (*62*, *63*) or on 3D analyses of freshly processed human cortical specimens up to 1-mm thick using antibody-based labeling approaches (*24*, *64*). Larger-volume 3D analysis of freshly processed human cortex up to 1 cm thick has been achieved using small-molecule dyes that readily penetrate thick tissue, but this labeling approach cannot distinguish neurons from other cell types (*16*, *17*). In our study, anti-NeuN immunolabeling and a largely automated data analysis pipeline enabled the assessment of neuronal density in large archival FFPE human cortex specimens, allowing detailed 3D profiling of neuronal distribution across distinct cortical layers. We applied this pipeline to a case series of FCD, a neurodevelopmental malformation disorder characterized by subtle and focal pathology that often challenges diagnostic assessment by conventional 2D histology. The 3D cortical profiling reflected the distortion of cortical layer architecture in FCD. Furthermore, cell volume analysis detected the large, rounded balloon cells characteristic of the most prevalent FCD subtype IIb, facilitating FCD diagnosis. Moreover, we observed multiple foci of neuronal loss, likely resulting from the long-lasting epilepsy with excitotoxic neuronal injury in neuronal assemblies. Previously, only two studies have reported neuronal loss in FCD using conventional 2D histology (*65*, *66*). However, the extent of neuronal loss in the entire affected cortex had not been determined. aDISCO-based quantitative 3D histology revealed that the lesional foci constituted only a minor fraction of the cortical volume in whole-mount FFPE tissue blocks, a level of sparsity that conventional 2D histology is prone to overlook. Furthermore, there was additional neuronal loss not visible by eye and only detectable via quantification of neuronal cell densities. Together, these findings underscore the importance of quantitative 3D histology for holistic examination of human tissue specimens and promise to entail direct implications for diagnostic quality in FCD.

The primary current limitation of aDISCO is the relatively long, multistep protocol that prolongs processing time compared to conventional 2D histology. For this reason, aDISCO is best suited for retrospective 3D human histological studies and as a complementary diagnostic tool in particularly challenging cases, rather than for rapid routine histological diagnostics. Another consideration is the substantial data volume generated by high-magnification 3D light-sheet imaging of large human tissue blocks on the terabyte scale. Managing, storing, and analyzing these data require appropriate computational infrastructure and expertise. Last, a general limitation of human studies compared to genetically and environmentally controlled animal models is the considerable interindividual variability between patients. While this variability can complicate interpretation, statistically significant findings in human samples may hold greater translational relevance as they reflect authentic clinical heterogeneity.

In conclusion, human tissue clearing with aDISCO enables 3D histology across a broad range of organs and cell types in whole-mount archival human tissue blocks, previously inaccessible due to long-term FFPE storage. In combination with 3D data analysis, aDISCO allows quantitative 3D histology to address biological and pathological questions, including rare diseases, owing to the availability of human samples collected over years to decades. aDISCO’s broad applicability, compatibility with diverse antibodies, and integration with automated 3D data analysis can contribute to improved histological diagnostics and deeper insights into human diseases.

## MATERIALS AND METHODS

### Ethics statement

All procedures and the associated guidelines for processing archival FFPE human tissue were approved by the cantonal ethics committee Zurich (BASEC no. 2022-00293). Because of anonymization and because a consent to participate could not be obtained after long-term sample storage of diagnostic surplus material, the study was approved without the need for specific patient consent.

### Archival human samples

FFPE human tissue blocks were selected from the surgical and autopsy diagnostic archive at the Institute of Neuropathology and the Institute of Pathology and Molecular Pathology, University Hospital Zurich, from cases between the years 2006 and 2011. All samples were processed between 2019 and 2023. The samples for testing compatibility of the aDISCO protocol with CNS regions and human organs originated from autopsies and were selected in an anonymized way. For the use-case study, FFPE brain surgical specimens from seven FCD patients were chosen from the diagnostic archive and pseudonymized. Patients with FCD were selected on the basis of available clinical data and neuropathological diagnosis from conventional 2D histology. The hospital software database “KISIM” was used to check the medical history of patients with FCD. Autopsy samples from seven age-matched patients with normal histology in the respective cerebral lobes served as controls. For controls, only basic demographic information and brain location were available due to pseudonymization and associated ethical restrictions. Exclusion criteria for controls encompassed any pathologies in the respective brain region.

### Deparaffinization of whole-mount archival human tissue blocks

FFPE human tissue blocks were melted for one hour at 60°C in a tissue embedding system TES99 (Medite). Samples were transferred into glass vials with closed top cap (C226-0020, Thermo Fisher Scientific). The remaining wax was removed by incubation in xylene (253-VL51TE, Thommen-Furler AG) two times each for 1 hour at 37°C, shaking at 50 rpm. Samples were rehydrated in decreasing serial solutions (100, 95, 90, 80, 70, 50, and 25%) of ethanol (20821.321, VWR Chemicals) in Milli-Q water (Merck Millipore) each for 1 hour at room temperature, shaking at 50 rpm, and lastly in 1× phosphate-buffered saline (PBS) overnight at room temperature, shaking at 50 rpm.

### Dehydration and preclearing

Samples were dehydrated in increasing serial solutions (20, 40, 60, and 80%) of THF (186562, Sigma-Aldrich) in Milli-Q water each for 1 hour at room temperature, shaking at 50 rpm, followed by two times 100% THF each for 1 hour at room temperature, shaking at 50 rpm. Preclearing was performed in a 2:1 solution of DCM (270997, Sigma-Aldrich) to THF overnight at room temperature, shaking at 50 rpm.

### Rehydration and bleaching

DCM was washed out by incubating the samples two times in 100% THF each for 30 min at room temperature, shaking at 50 rpm. Rehydration was achieved in decreasing serial solutions (80, 60, and 40%) of THF in Milli-Q water each for 1 hour at room temperature, shaking at 50 rpm. To precool the samples for bleaching, the rehydration step in 20% THF in Milli-Q water was performed for 1 hour at 4°C, shaking at 50 rpm. Bleaching was conducted in 28% H_2_O_2_ (1.08600.2500, Merck Millipore) with 4% THF in Milli-Q water for 20 hours at 4°C in the dark, shaking at 50 rpm.

### Autofluorescence removal

To wash out the bleaching solution, samples were incubated two times in 1× PBS each for 1 hour at room temperature, shaking at 50 rpm. To remove remaining autofluorescence from highly pigmented samples, tissue blocks were incubated in 10 mM cupric sulfate (61230, Fluka) in 50 mM ammonium acetate (A-7330, Sigma-Aldrich) buffer in Milli-Q water, pH = 5, for 3 hours at room temperature in the dark, shaking at 50 rpm. The solution was washed out by subsequent incubation in 1× PBS two times each for 1 hour and then overnight at room temperature, shaking at 50 rpm.

### Antigen retrieval

Depending on the respective antibodies (table S2), antigen retrieval was achieved by treating the samples with 10 to 30% formic acid (4724.3, Roth) solution in Milli-Q water (pH = 2) for 3 hours at room temperature, shaking at 50 rpm. The solution was washed out by incubation in 1× PBS for 1 hour at room temperature, shaking at 50 rpm.

To test different antigen retrieval reagents, large archival human brain tissue blocks were treated with 10% formic acid in Milli-Q water (pH = 2), 2 N hydrochloric acid (124630010, Acros Organics) in Milli-Q water (pH = 0.6), 0.05% trypsin (2193.3, Roth) + 0.1% calcium chloride (21115, Sigma-Aldrich) in Milli-Q water (pH = 7.8), or 0.002% proteinase K (7528.6, Roth) + 0.053% calcium chloride in TE buffer [50 mM tris (2449.2, Roth) + 1 mM EDTA (8043.2, Roth) + 0.5% Triton X-100 (X100, Sigma-Aldrich), pH = 8] for 3 hours at room temperature, shaking at 50 rpm. Solutions were washed out by incubation in 1× PBS for 1 hour at room temperature, shaking at 50 rpm.

### Permeabilization and decolorization

Samples were first permeabilized with 0.2% Triton X-100 in 1× PBS two times each for 1 hour at room temperature, shaking at 50 rpm. Stronger permeabilization and decolorization were achieved by subsequent incubation in 25% urea (U4884, Sigma-Aldrich) + 25% *N*,*N*,*N′*,*N′*-tetrakis(2-OH-propyl)ethylenediamine (122262, Sigma-Aldrich) + 15% Triton X-100 in 1× PBS overnight at 37°C, shaking at 50 rpm. Permeabilization was continued in 0.2% Triton X-100 + 2.3% glycine (G8898, Sigma-Aldrich) + 20% dimethyl sulfoxide (DMSO) (D8418, Sigma-Aldrich) + 0.5 mM methyl-beta-cyclodextrin (332615, Sigma-Aldrich) + 0.2% trans-1-acetyl-4-OH-l-proline (441562, Sigma-Aldrich) + 0.1% sodium azide (S8032, Sigma-Aldrich) in 1× PBS for 3 days at 37°C, shaking at 50 rpm.

### Blocking and immunolabeling

For blocking and antibody staining, samples were transferred from glass vials into 6-well (92006, TPP) or 12-well plates (92012, TPP), depending on sample size, and sealed with parafilm (10018130, Bemis) during incubation to avoid evaporation. Blocking was performed in 0.2% Tween 20 (P2287, Sigma-Aldrich) + 0.1% heparin (10 mg/ml; 2835358, B. Braun Medical AG) + 5% DMSO + 6% donkey serum (017-000-121, Jackson ImmunoResearch) in 1× PBS for 2 days at 37°C, shaking at 50 rpm. Immunolabeling was conducted in 0.2% Tween-20 + 0.1% heparin (10 mg/ml) + 5% DMSO + 0.1% sodium azide (staining buffer) for up to 4 weeks, depending on sample size and antibody diffusion capacity, at 37°C, shaking at 50 rpm. To enhance the diffusion capacity of antibodies, immunolabeling was carried out gradually, starting with low antibody concentrations and adding more and more antibodies to the staining solution to finally reach the calculated final antibody concentrations at the end of the staining time. For this gradual immunolabeling step, the starting and final antibody concentrations and the staining times were adapted to the specific antibodies used in this study (table S2). For example, when the final dilution of an antibody with a stock concentration of 1.5 mg/ml was 1:200 and the staining time was 2 weeks, we started with a dilution of 1:800 and added the same dilution another three times every half a week, thus reaching the final dilution of 1:200 after 2 weeks.

Between primary and secondary antibody staining, samples were washed in staining buffer two times each for 1 hour, then two times each for 2 hours, and then overnight at room temperature, shaking at 50 rpm. After secondary antibody incubation, samples were washed in staining buffer two times each for 1 hour, then two times each for 2 hours, and then for 2 days at room temperature, shaking at 50 rpm.

### Dehydration and clearing

Samples were dehydrated in increasing serial solutions (20, 40, 60, and 80%) of THF in Milli-Q water each for 1 hour at room temperature, shaking at 50 rpm, followed by two times 100% THF each for 1 hour at room temperature, shaking at 50 rpm. Final delipidation was performed in a 2:1 solution of DCM to THF overnight at room temperature, shaking at 50 rpm, followed by incubation in 100% DCM two times each for 2 hours at room temperature, shaking at 50 rpm. RI (*n*_D_ = 1.56) matching was achieved in dibenzyl ether (DBE) (108014, Sigma-Aldrich) overnight at room temperature, nonshaking.

### Assessment of aDISCO clearing-induced tissue shrinkage

Two orthogonal sample dimensions (*x* and *y*) were measured from macroscopic images acquired in the rehydrated state following deparaffinization and again after final clearing with RI matching. Tissue shrinkage for each sample was quantified as the geometric mean of the relative dimensional changes along the *x* and *y* axes.

### Reparaffinization of cleared tissue blocks

DBE was washed out by incubating the tissue blocks in 100% methanol (203-VL03K-/1, Thommen-Furler AG) two times each for 1 hour at room temperature, shaking at 50 rpm. Samples were rehydrated in decreasing serial solutions (80, 60, 40, and 20%) of methanol in Milli-Q water each for 1 hour at room temperature, shaking at 50 rpm, followed by incubation in 1× PBS overnight at room temperature, shaking at 50 rpm. Tissue blocks were again dehydrated in increasing serial solutions (25, 50, and 70%) of ethanol in Milli-Q water each for 1 hour at room temperature, shaking at 50 rpm. Then, samples were put in the Leica ASP300S Fully Enclosed Tissue Processor (Leica Biosystems), and the following program started: 100% ethanol for 45 min, 90 min at 37°C, followed by 100% ethanol two times each for 1 hour at 45°C; then xylene for 45 min and two times each for 1 hour at 37°C; and lastly paraffin (39601006, Leica Biosystems) for 75 min, 90 min, and 2 hours at 62°C.

### 2D histology

Tissue sections (2-μm thick) were cut from the tissue blocks, using a semi-automated rotary microtome (RM2245, Leica Biosystems) and mounted onto slides (0810531, HistoBond). Slides were deparaffinized by incubation in xylene two times each for 10 min at room temperature. Slides were rehydrated in decreasing serial solutions (100, 95, 90, 80, and 70%) of ethanol in Milli-Q water each for 10 min at room temperature, followed by pure Milli-Q water for 10 min at room temperature.

For hematoxylin and eosin staining, sections were first incubated in filtered hematoxylin Harris (2E-056, Waldeck) for 5 min at room temperature, followed by rinsing in Milli-Q water. Sections were differentiated in 0.185% hydrochloric acid in Milli-Q water by four quick immersions at room temperature, followed by rinsing in tap water. Bluing was performed in 0.015% ammonia water (1.05432.1000, Merck Millipore) by six quick immersions at room temperature, followed by rinsing in Milli-Q water, and four quick immersions in 70% ethanol at room temperature. Sections were incubated in 0.075% eosin (1B-425, Waldeck) + 0.0075% phloxine (15926.0025, Merck Millipore) + 0.5% acetic acid (1.00063.1011, Merck Millipore) + 84% ethanol in Milli-Q water for 3 min at room temperature. After rinsing in 100% ethanol and xylene, they were lastly covered with coverslips (01012227, Biosystems).

For antibody staining, a Ventana Bench Mark Ultra Plus fully automated staining machine (Roche) was used, containing the Bench Mark Ultra LCS staining kit. Antigen retrieval was achieved with heated tris-borate-EDTA buffer in Milli-Q water (pH = 8) for 45 min, followed by rinsing in Milli-Q water (Ventana CC1 program). Staining was visualized with horseradish peroxidase and 3,3′-diaminobenzidine, included in the kit. Slides were scanned using a Hamamatsu C9600 digital slide scanner (Hamamatsu Photonics).

### 3D light-sheet imaging

Light-sheet imaging was performed using a custom-built mesoSPIM V5 (*30*). For all image settings, the following lasers and filter combinations were applied: 488-nm laser with a 520/35 BrightLine hard-coated filter (AHF Analysentechnik AG, part number #F37-520SG) or QuadLine Rejectionband ZET405/488/561/640 filter (AHF Analysentechnik AG, part number #F57-405SG); 561-nm laser with 561 Razor Edge long-pass (LP) filter (AHF Analysentechnik AG, part number #F76–561) or QuadLine Rejectionband ZET405/488/561/640 filter; 640-nm laser with 647 razor edge LP filter (AHF Analysentechnik AG, part number #F76-647); and 647-nm laser with QuadLine Rejectionband ZET405/488/561/640 filter. Laser intensities were individually set for the samples/each experiment to achieve the best SNR. For instance, for the autofluorescence removal experiment, the same settings were used for all samples. For overview whole-mount 3D images, the 1× objective was used at zoom levels of 0.63×, 1×, or 2×, depending on sample size. Samples were imaged in a 40 mm–by–40 mm–by–40 mm cuvette filled with DBE (*n*_D_ = 1.56), and *z*-step size was chosen to reach (near) isotropic voxel size. Camera exposure time was 10 ms.

For the use-case study, tile scanning of each entire cortex sample was performed with the 1× objective at 4× zoom. Samples were imaged in a 30 mm–by–30 mm–by–30 mm cuvette filled with DBE, with a *z*-step size of 1 μm. Camera exposure time was 10 ms.

For high-magnification ROI imaging of almost all samples, a 2× objective (MVPLAPO2XC, Olympus/Evident) was used in combination with a dipping cap (205915, LaVision BioTec). The front cover glass of the dipping cap was removed and replaced with a 40 mm–by–40 mm–by–40 mm cuvette (Portmann Instruments) filled with DBE. This objective was used at zoom levels of 2×, 4×, or 6.3×, and *z*-step size was chosen to reach (near) isotropic voxel size. With this system at highest zoom level (6.3×) and a numerical aperture (NA) of ∼0.2, the standard Rayleigh criterion for the lateral (*xy*) diffraction-limited resolution at 561-nm wavelength is

$$\delta \mathrm{xy}=\frac{0.61\;\lambda }{\text{NA}}=\frac{0.61\times 561}{0.2}=1710\;\text{nm}$$

Camera exposure time was 10 ms.

Alternatively, for the peripheral nerve sample, a modified detection path based on the Benchtop mesoSPIM (V6) with a Mitutoyo objective turret (Mitutoyo) and a Mitutoyo G Plan Apo 20× objective was used (*31*). Samples were imaged in a 40 mm–by–40 mm–by–40 mm cuvette filled with DBE (*n*_D_ = 1.56), and *z*-step size was chosen to reach (near) isotropic voxel size. Camera exposure time was 20 ms.

### Qualitative image processing

For 3D data analysis, an AMD Ryzen W137 Threadripper PRO Workstation with 2x NVIDIA Geforce RTX 3090 24 Gigabytes (GB) Graphics Double Data Rate (GDDR)6X graphic cards (Brentford) was used. Fiji software [64 bit, version 2.3.0/1.53f51 (*67*)] was used for qualitative image processing, including cropping, contrast adjustment, background subtraction, contrast limited adaptive histogram equalization (CLAHE), color and look-up-table intensity assignment, channel merging, generation of hyperstacks with temporal color code, generation of substacks and maximum intensity projections, generation of movies, and scale bar assignments. Display ranges were set such that background regions outside the sample mapped to black. The volume viewer plugin (*68*) was applied for volume rendering. The 3DScript plugin (*69*) was used for volume rendering and for generating complex movie sequences via encoded movie scripts. Transparency was altered by adapting the alpha value. Adobe Premiere Elements (Adobe version 2021) was applied for further image and movie processing, including sharpening, brightening, movie clipping, and text labeling.

### Quality assessment of light-sheet imaging data

#### Fourier ring correlation quality estimate

Fourier ring correlation quality estimate (FRC-QE) was conducted using a published Fiji package (*37*) with the default parameters.

#### Dendritic cross-sectional profiles

Dendrites with small diameter were visually selected and a cross-sectional profile of 5 to 10 pixel width drawn using the *straight line* tool in Fiji. Values were saved in a csv-file and imported to MATLAB (MathWorks, version 2022b) for Gaussian fitting with the *cftool* toolbox. The full width of half maximum was calculated from the SD of the fitted Gaussian.

#### Tracing of neuronal processes

Main processes of example dopaminergic neurons were traced using the *brush* tool of the tracing framework webKnossos (*38*) in the Web interface.

#### MFI projection and z-profile

Codes for the assessment of MFI projection and *z*-profile were written in MATLAB. For both calculations, the background of the sample was excluded by setting all background pixels without sample to not-a-number. The MFI projection was calculated as the mean projection value along the *z* axis and plotted as heatmap in *xy* direction. The *z*-profile was calculated as the MFI for each imaging plane (in *z*) across the sample, with the SEM computed across the pixels of each plane.

### Stitching

3D image tiles were automatically stitched via a custom-written code in R studio [version 1.2.1335, R version 3.6.0 (*70*)] using Fiji and TeraStitcher [version 1.11.6. (*71*)].

### Neuronal cell segmentation and detection

To automatically and reliably detect neurons in human brain cortex, a three-step procedure was implemented in Python (versions 2.7. and 3.10.12 (*72*, *73*)). First, stitched raw stacks were segmented using a supervised random forest machine learning classifier (ILASTIK version 1.4.0rc6 (*40*)) into coherent 3D particles that were then identified as initial neuronal cell candidates using an established cell detection pipeline [ClearMap 1.0 (*41*)]. To train the ILASTIK classifier, ground truth was manually annotated volumetrically as foreground versus background in 13 representative substacks of 200 × 200 × 200 voxels. Representative substacks were generated from the entire 15-TB sized dataset, representing various regions (transitions of different cortical layers and white matter, regions with high and lower SNR) from different samples (controls and patients with FCD). To fit the input into the memory for ILASTIK processing, the ClearMap pipeline was parallelized. Segmentation was performed such that overdetection was more likely than underdetection to enable subsequent refinement by discarding false positives. The refinement of cell candidates is conceptually similar to an existing pipeline (*74*) for murine cell detection but has been implemented independently here for our human data. Initial neuronal cell candidates were detected based on local intensity maxima using ClearMap. For quality checks, results were visualized in Fiji. Second, initial cell candidates were merged using a custom-written 3D-peak finder based on 3D gradient ascent. Briefly, for each initial neuronal cell candidate determined by ClearMap in step 1, the local raw 3D image (31 × 31 × 91 voxels in *xyz*) was extracted and smoothed with a Gaussian filter (SD of 1.0, 1.0, and 2.5 pixels in *x*, *y*, and *z* direction, respectively). Within this local environment, the position of the detected cell was iteratively optimized to follow the maximum image intensity until the algorithm converged to a local maximum. Upon this gradient-based refinement of neuron positions, cells that ended up at the same maximum were merged. Code for gradient-based refinement and merging of neuronal cell candidates is available on GitHub (https://github.com/PTRRupprecht/Cell_Detection/tree/main/Gradient_cell_merger). Third, the refined neuronal cell candidates were classified as cells or noncells using a DCNN-based binary classifier. This procedure served to exclude non-neuronal somata bright structures such as proximal axons, hot pixels of the camera sensor, or spurious noise. Briefly, each 100 neuronal cell candidates as output from the second processing step were chosen at random from 19 different 3D samples, resulting in a total of 1900 neuronal cell candidates for ground truth annotation and validation. These neuronal cell candidates were manually annotated by visually inspecting *xy* sections (31 × 31 pixels) and *xz* sections (31 × 91 pixels), centered at the location of the candidate cell. Each neuronal cell candidate was assigned either a “cell” or “noncell” label. A Python-based interactive tool used to perform these annotations is available on GitHub (https://github.com/PTRRupprecht/Cell_Detection/tree/main/Label_tool). To assess annotation reliability, the dataset was re-annotated by the same annotator (intra-rater) and independently by a second annotator (inter-rater). Agreement between annotations was high (intra-/inter-rater: accuracy of 91%/91%, precision of 97%/92%, recall of 91%/91%, *F*_1_ score of 94%/91%). Next, DCNNs were trained on the basis of the annotated ground truth to predict whether a detected neuronal cell candidate was a cell. Three networks for different sections (*xy*, *xz*, and *yz*) were combined by adding their outputs. All networks were standard convolutional networks with two 2D convolutional layers with rectified linear unit (ReLU) functions, followed each by a 2 × 2 max pooling layer, a subsequent set of two dense layers with ReLU functions, and a final layer with two neurons. These two output neurons represented the two classes (cell and noncell). The network was defined in PyTorch (*75*) and trained with a standard Adagrad (*76*) optimizer using cross-entropy loss with a batch size of 10. The classification performance plateaued after approximately 1500 training epochs (fig. S8C). Randomly selected neuronal cell candidates (1800) were used for training. Performance was evaluated using cross-validation with randomly selected withheld neuronal cell candidates (100 random cell candidates withheld of a total of 1900). To improve cell classification, averaging across deep network ensembles was used (*77*). Five instantiations of each network type (*xy*, *xz*, and *yz*) were trained and their predictions were linearly combined for ensemble averaging across 15 models. On the basis of ensemble predictions, neuronal cell candidates were accepted or discarded. In cross-validated performance evaluation, the classifier was correct for 92.2% of neuronal cell candidates and incorrect for 7.8%, with false positives and false negatives at 4.2 and 3.6%, respectively. Python code to train and apply the neuronal cell candidate classifier together with an example ground truth dataset of manually annotated neuronal cell candidates is available on GitHub (https://github.com/PTRRupprecht/Cell_Detection/tree/main/Binary_classification_neuron_candidates).

### Quantification of neuronal density

To quantify neuronal density, neurons in the 3D stack were detected as described above, yielding the total number of detected neurons. Subsequently, the number of detected neurons was divided by the volume occupied by the brain sample. The volume of the brain sample was obtained by a binary 3D mask computed in Python using a manually defined global threshold. The resulting 3D mask was corrected and refined by manual annotation of the 12×-downsampled segmented stack in 3D with three interactive views (*xy*, *xz*, and *yz*) using ITK-SNAP [version 3.6.0 (*78*)]. To distinguish cortical versus white matter brain volumes, cortical regions of 12×-downsampled imaging stacks were manually annotated in 3D with three interactive views (*xy*, *xz*, and *yz*) using ITK-SNAP (version 3.6.0). The manual annotation of focal neuronal loss regions was performed in ITK-SNAP (version 4.0.1) by carefully inspecting the 4×-downsampled brain tissue blocks in 3D with three interactive views (*xy*, *xz*, and *yz*).

### Neuronal cell density profiles across cortical layers

To assign “cortical depth” normalized to each position in a stack by the local cortical thickness, landmarks within the cortical sample were annotated, hyperplane fits of these annotations applied, and the distance between these hyperplanes scaled to normalize for cortical thickness. First, to annotate landmarks, we identified the outer brain boundary, the boundary between the cortical layers I and II, the stripe of increased neuronal density in layer IV, and the boundary between layer VI and the white matter. Sections in the *xy* plane were manually annotated with five to eight coordinates for the respective landmark in a custom-written interactive Python script. Every 100 pixels (100 μm) in the *z* direction, an *xy* section was annotated, and a third-order polynomial hyperplane was fitted to each of the landmark layers. For each neuron in the 3D volume, the shortest distance to each of the landmark hyperplanes was determined, which enabled the computation of the local relative distance between two landmark layers.

### Figure design

Figures were designed in Affinity Designer 2 (v2.3.1) and Adobe Illustrator (v2025). Computer-aided design (CAD) models were designed in Autodesk Inventor (v2023).

### Statistics

Statistical analysis (Fig. 7M) was performed by permutation test, which avoids assumptions regarding data normality.
