# Supplementary material for: aDISCO: A clearing method to enable 3D microscopy of large archival paraffin-embedded human tissue blocks
Source: Sci Adv. 2026 Aug 12;12(33):eaef9926. doi: 10.1126/sciadv.aef9926 (PMC13464637; doi:10.1126/sciadv.aef9926)
Supplement: Supplementary file 1 [file sciadv.aef9926_sm.pdf]

Science Advances
AAAS

Supplementary Materials for

aDISCO: A clearing method to enable 3D microscopy of large archival paraffin-embedded human tissue blocks

Anna Maria Reuss *et al.*

Corresponding author: Anna Maria Reuss, annamaria.reuss@uzh.ch; Peter Rupprecht, rupprecht@hifo.uzh.ch; Adriano Aguzzi, adriano.aguzzi@isab.science

Sci. Adv. **12**, eaef9926 (2026)
DOI: 10.1126/sciadv.aef9926

The PDF file includes:

Figs. S1 to S8
Tables S1 to S6
Legends for movies S1 to S7

Other Supplementary Material for this manuscript includes the following:

Movies S1 to S7

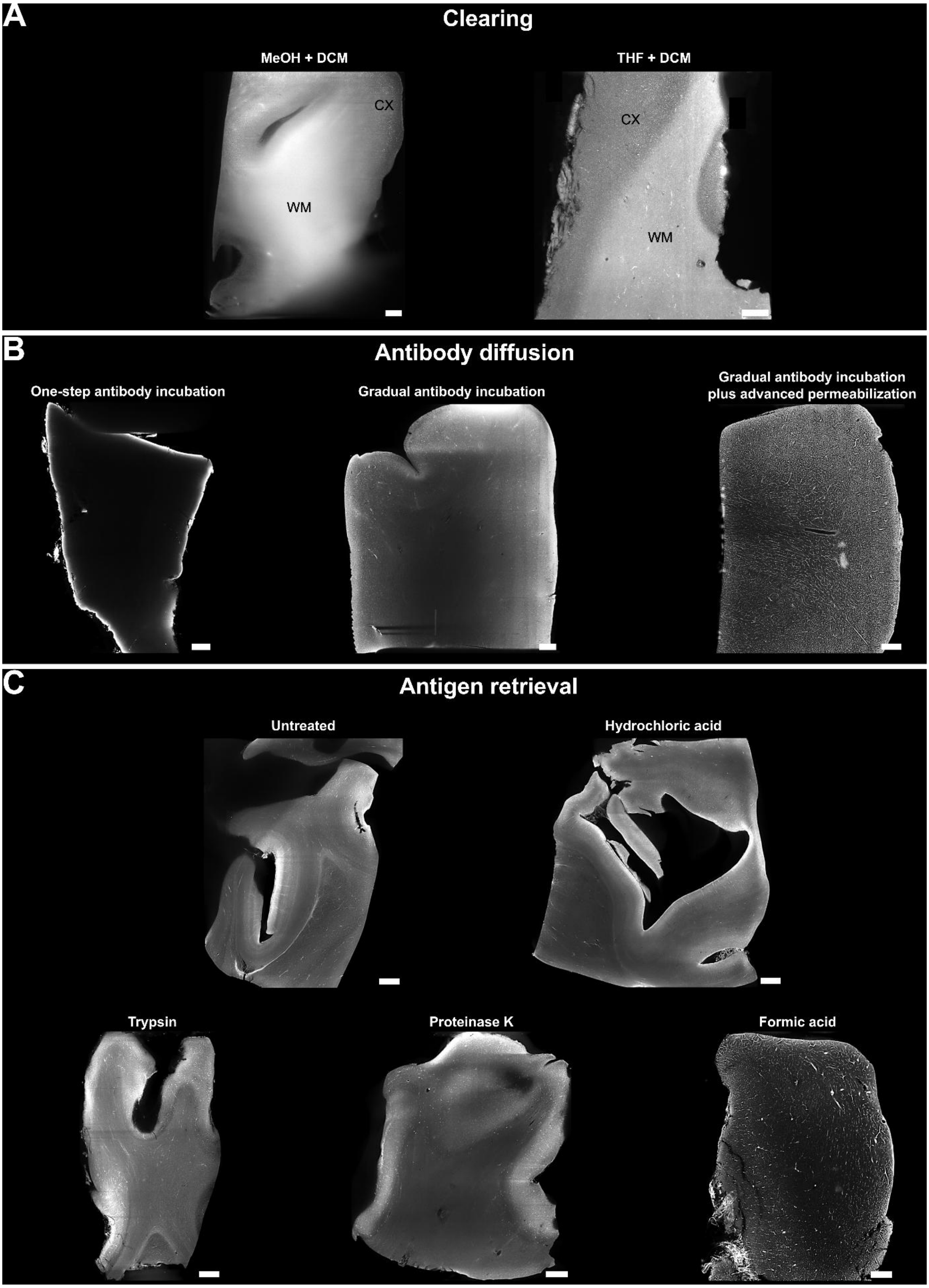

Fig. S1. aDISCO development in archival human brain cortex.

(A) Harsh clearing conditions, including substitution of methanol (MeOH) with tetrahydrofuran (THF), achieved effective clearing, especially of lipid-rich brain white matter (WM) relative to cortex (CX). **(B)** Optimization of anti-CD13 antibody diffusion by gradual immunolabeling and advanced permeabilization. **(C)** Compared to untreated control, anti-podocalyxin vessel staining signal is slightly enhanced by trypsin and significantly enhanced by formic acid, while it is reduced by hydrochloric acid and proteinase K treatment. Scale bars (A-C), 1 mm.

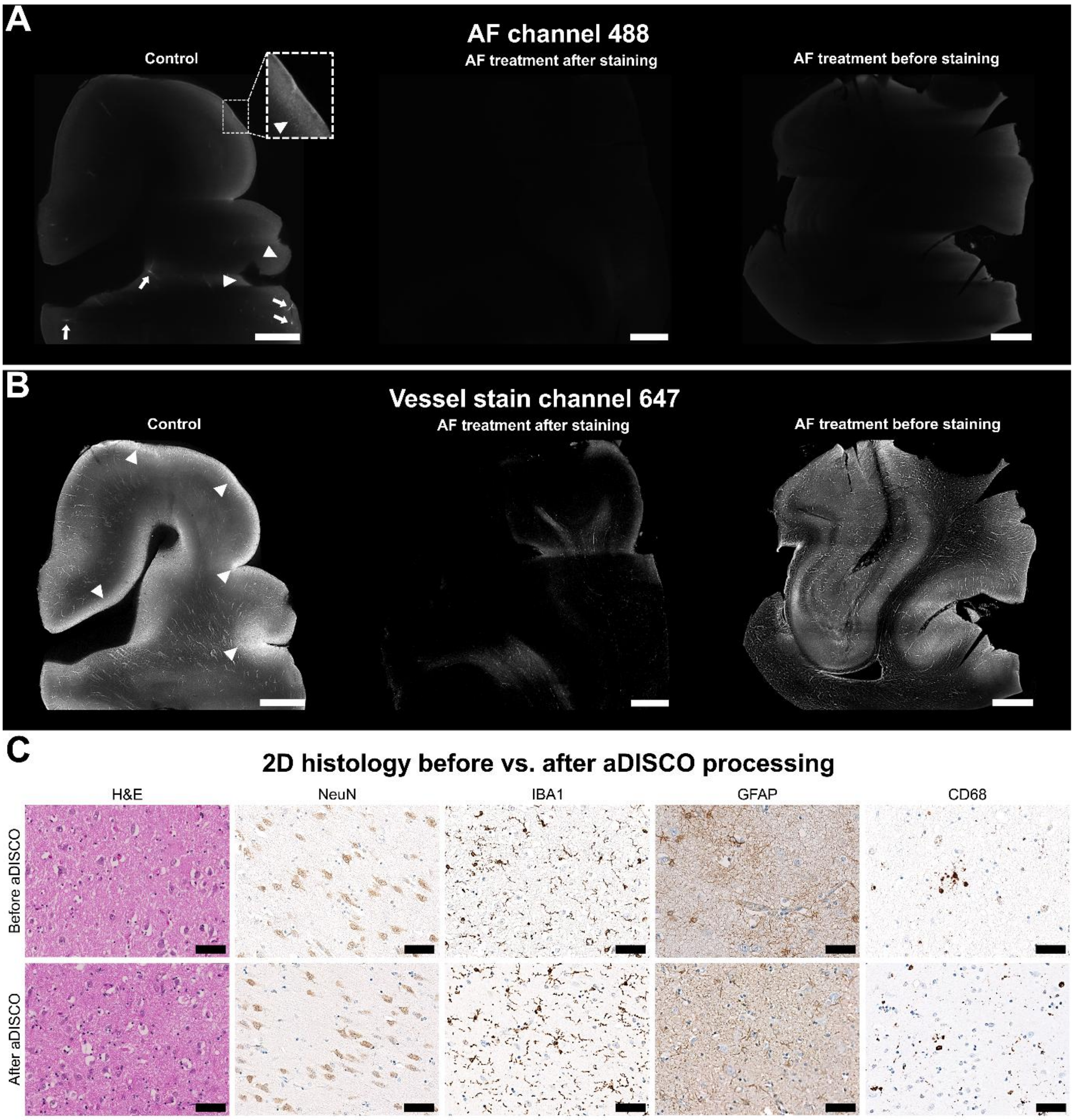

Fig. S2. Autofluorescence (AF) removal and reparaffinization.

(A) Unstained AF channel (488 nm) of archival human brain samples shows autofluorescent artifacts in the control sample, arising from vessels with coagulated blood (arrows) and lipofuscin (arrowheads) despite bleaching with 28% hydrogen peroxide. AF treatment applied either before or after staining removes these artifacts. **(B)** Vessel channel (647 nm) shows CD13-immunolabeled vasculature. In the control sample, lipofuscin autofluorescence interferes with the vessel signal (arrowheads). AF treatment after staining removes these artifacts but also substantially reduces the vessel signal, whereas treatment before staining preserves it. All samples (A-B) were imaged at 0.63x magnification and processed with identical settings. Scale bars, 2 mm. (**C**) Conventional 2D histology before vs. after aDISCO processing. 2-µm-thin sections cut from the same human brain FFPE tissue block before and after performing the entire aDISCO protocol with reparaffinization after tissue clearing. Sections were stained for hematoxylin and eosin (H&E) as well as for different antibodies, and imaged using a Hamamatsu C9600 digital slide scanner. Scale bars, 50 µm.

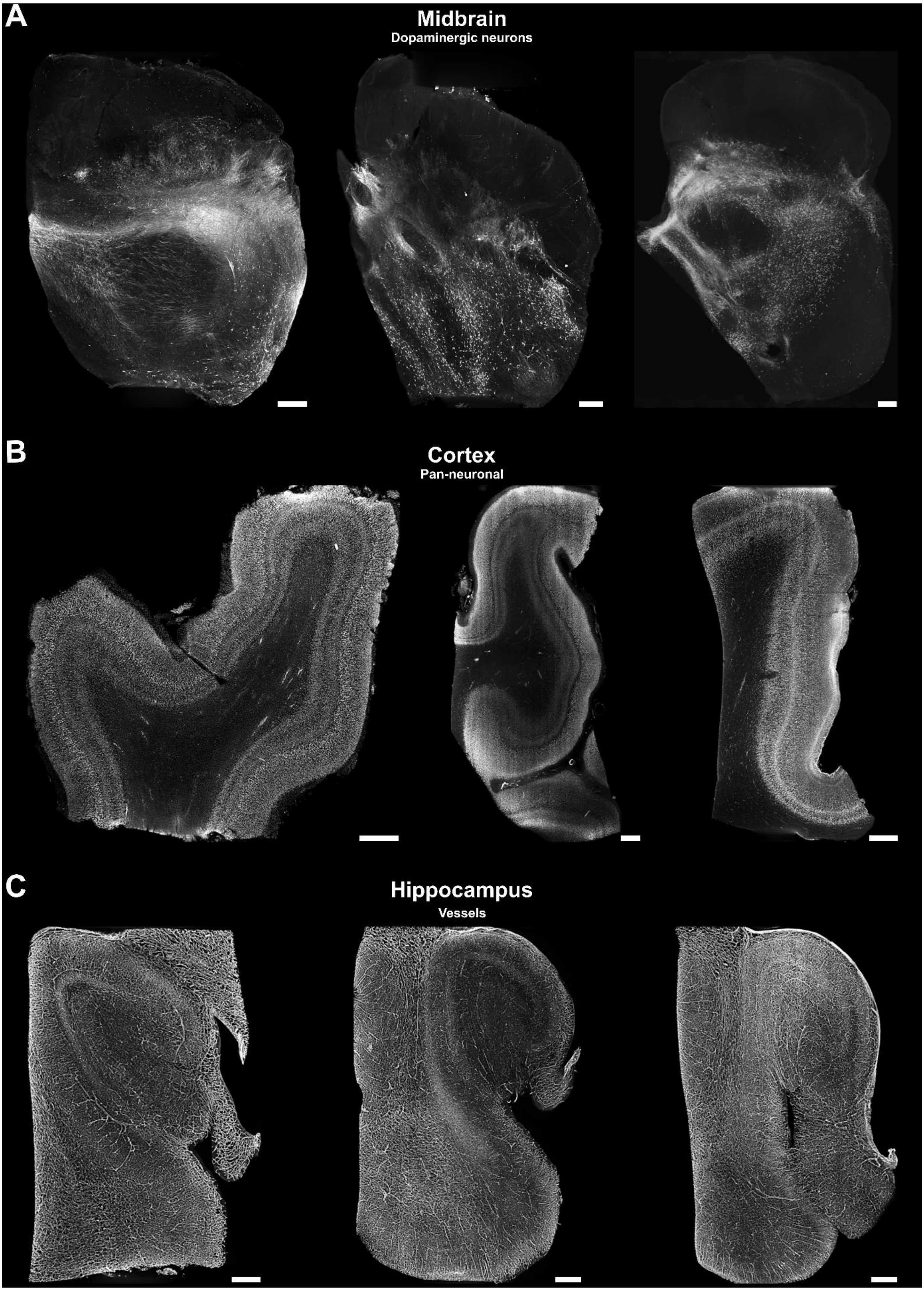

Fig. S3. Reproducibility of aDISCO in different human CNS regions.

Each panel presents a maximum intensity projection of 50 slices from the sample center (range: 425-980 slices, 5-10 µm *z*-step size) from individual patients. **(A)** Dopaminergic neurons (anti-tyrosine hydroxylase) in archival human midbrain. $N = 3$. **(B)** Pan-neuronal staining (anti-NeuN) in archival human brain cortex. $N = 3$. **(C)** Blood vessels (anti-podocalyxin) in archival human hippocampus. $N = 3$. Scale bars (A-C), 1 mm.

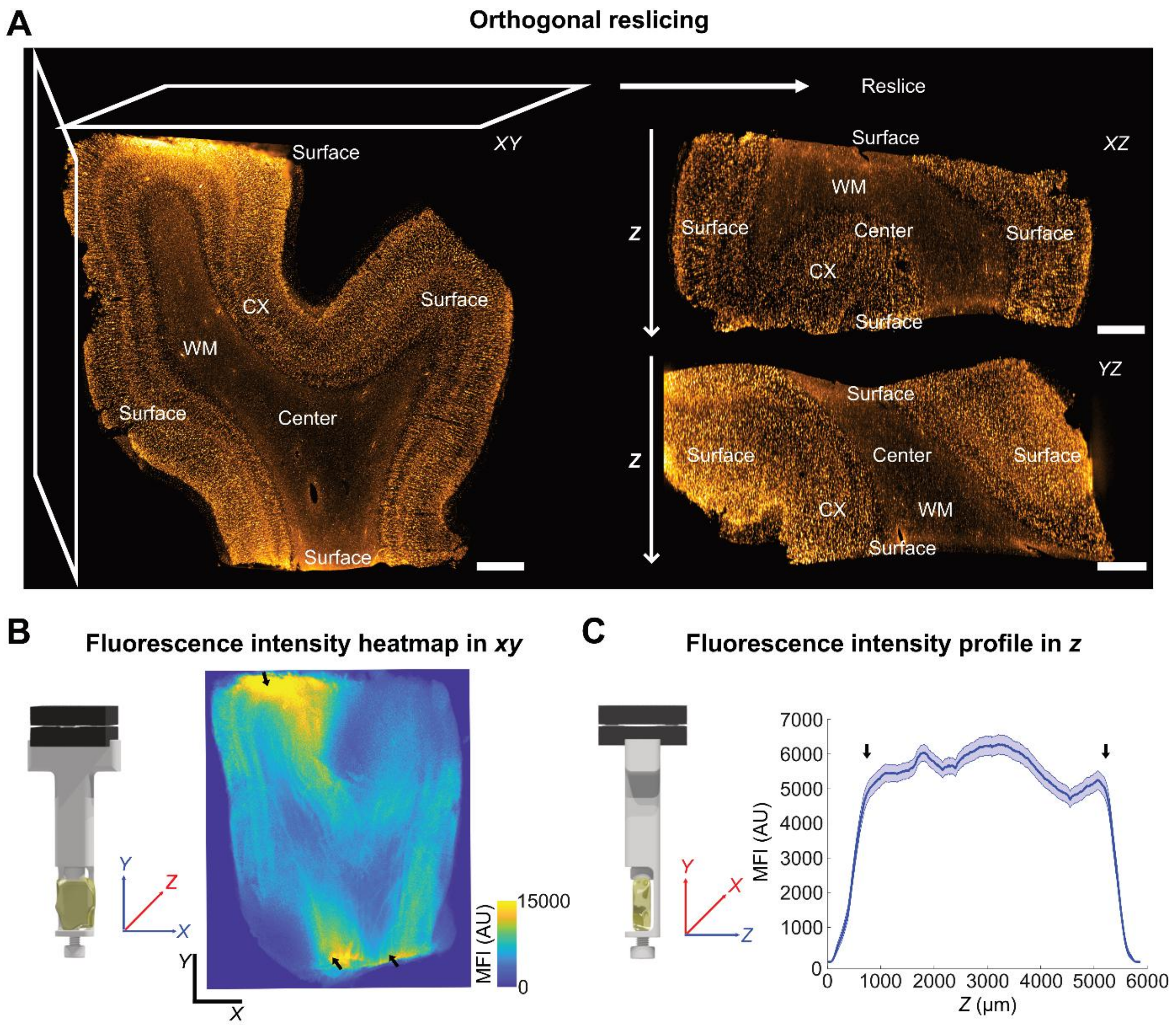

Fig. S4. Effective clearing and immunolabeling of aDISCO-processed archival human brain cortex.
(**A**) Large archival human brain cortex sample, immunolabeled for neurons (anti-NeuN) and imaged at 1.25x magnification. Left: Slice in *xy*-projection from the sample center in *z*-direction. Right: Slice in *xz*- (top) and *yz*-projection (bottom) from the sample center in *y*- and *x*-direction, respectively, generated via reslicing of the 3D stack. In all three projections, continuous staining signals in the cortex (CX), from the surfaces to the center, as well as effective clearing of the entire sample, including the neuron-poor and lipid-rich white matter (WM), can be observed. This continuity through the entire sample thickness is particularly visible in the resliced *xz*- (top) and *yz*-projection (bottom) along the *z*-axis (*z* arrows). Scale bars, 1 mm. (**B**) Mean fluorescence intensity (MFI) projection averaged over all planes in *z* (red axis) and plotted as heatmap in *xy* (blue axes), as illustrated by the computer-aided design (CAD) model. Focal high-intensity pixels at the top and bottom are due to screwing of the sample to the holder for imaging (black arrows). Color scale indicates MFI range in arbitrary units (AU). Scale bars, 2 mm. (**C**) *Z*-profile of MFI in AU, averaged over all *xy*-pixels (red axes) per imaging plane and plotted along the *z*-axis (blue) with standard error of the mean (light blue), as illustrated by the CAD model. Arrows indicate the beginning and end of the sample.

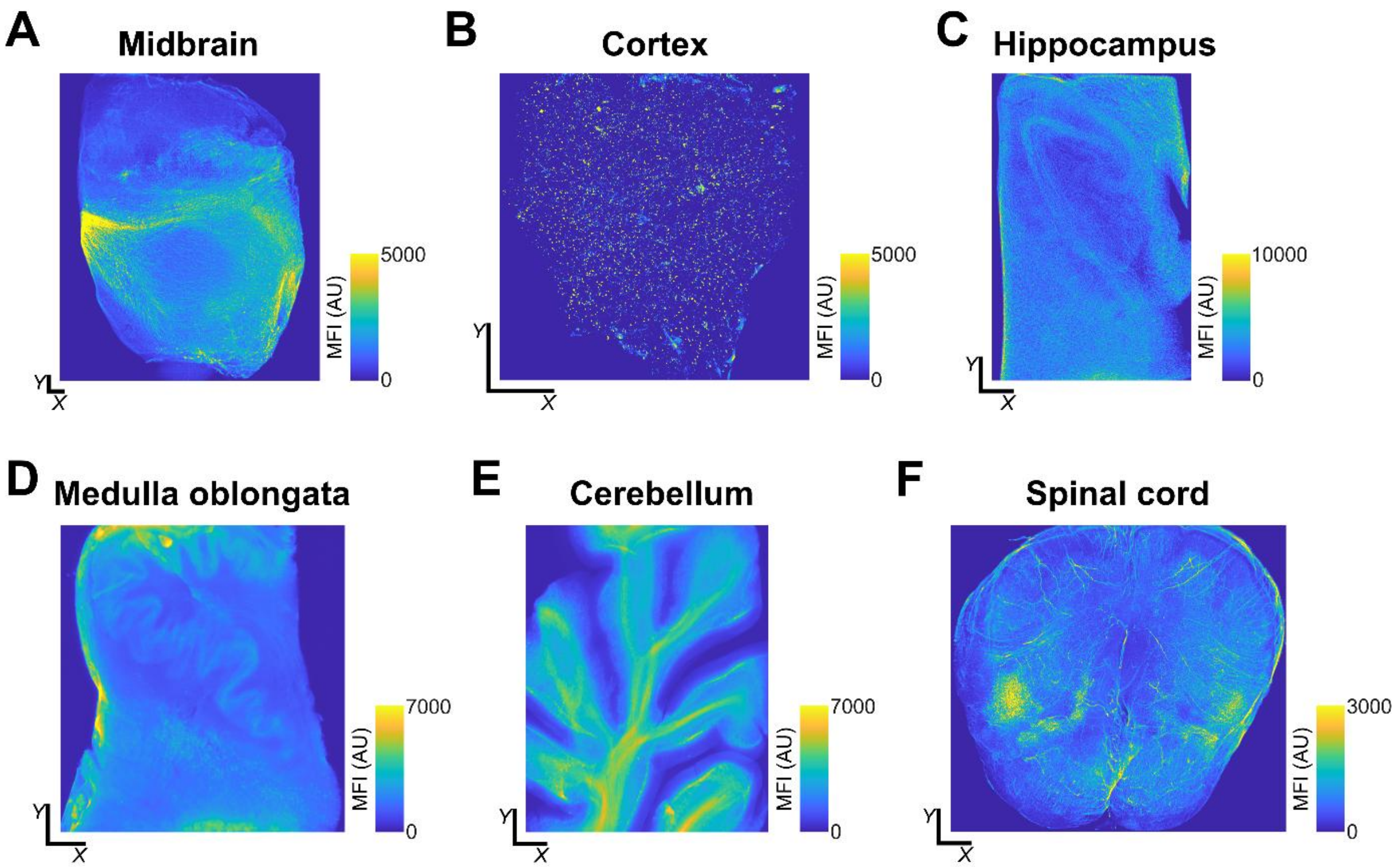

Fig. S5. Mean fluorescence intensity (MFI) projections of different archival human CNS regions.
(**A-F**) MFI projections averaged over all planes in *z* and plotted as heatmaps in *xy*. Color scales indicate MFI ranges in arbitrary units (AU). Scale bars, 1 mm.

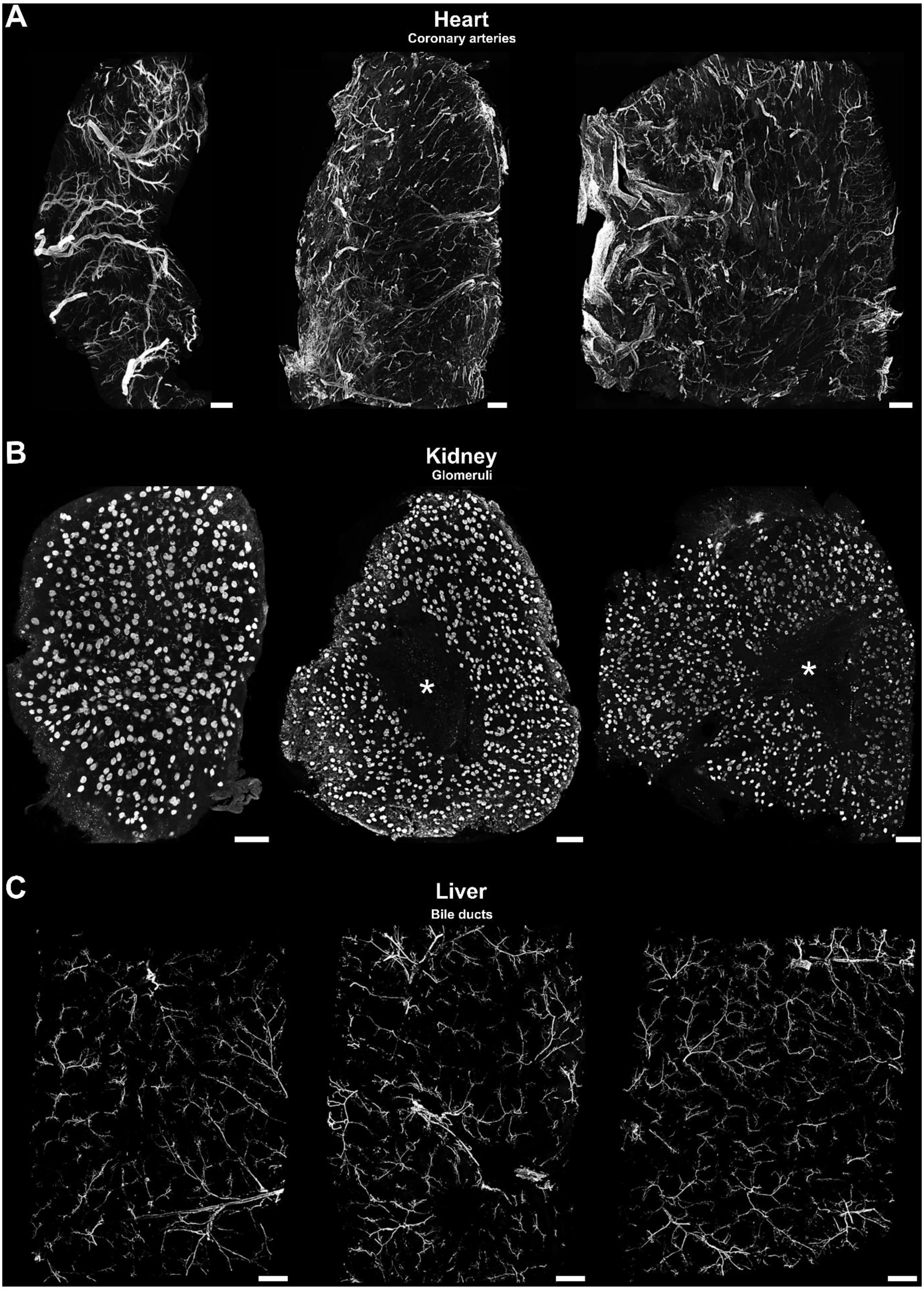

Fig. S6. Reproducibility of aDISCO in different human organs.

Each panel presents a maximum intensity projection of 50 slices from the sample center (range: 290-700 slices, 6-10 µm *z*-step size) from individual patients. **(A)** Coronary arteries (anti-SMA) in archival human heart. $N = 3$. **(B)** Glomeruli (anti-nephrin) in archival human renal cortex. *Renal medulla is spared. $N = 3$. **(C)** Bile ducts (anti-CK19) in archival human liver. $N = 3$. Scale bars (A-C), 1 mm.

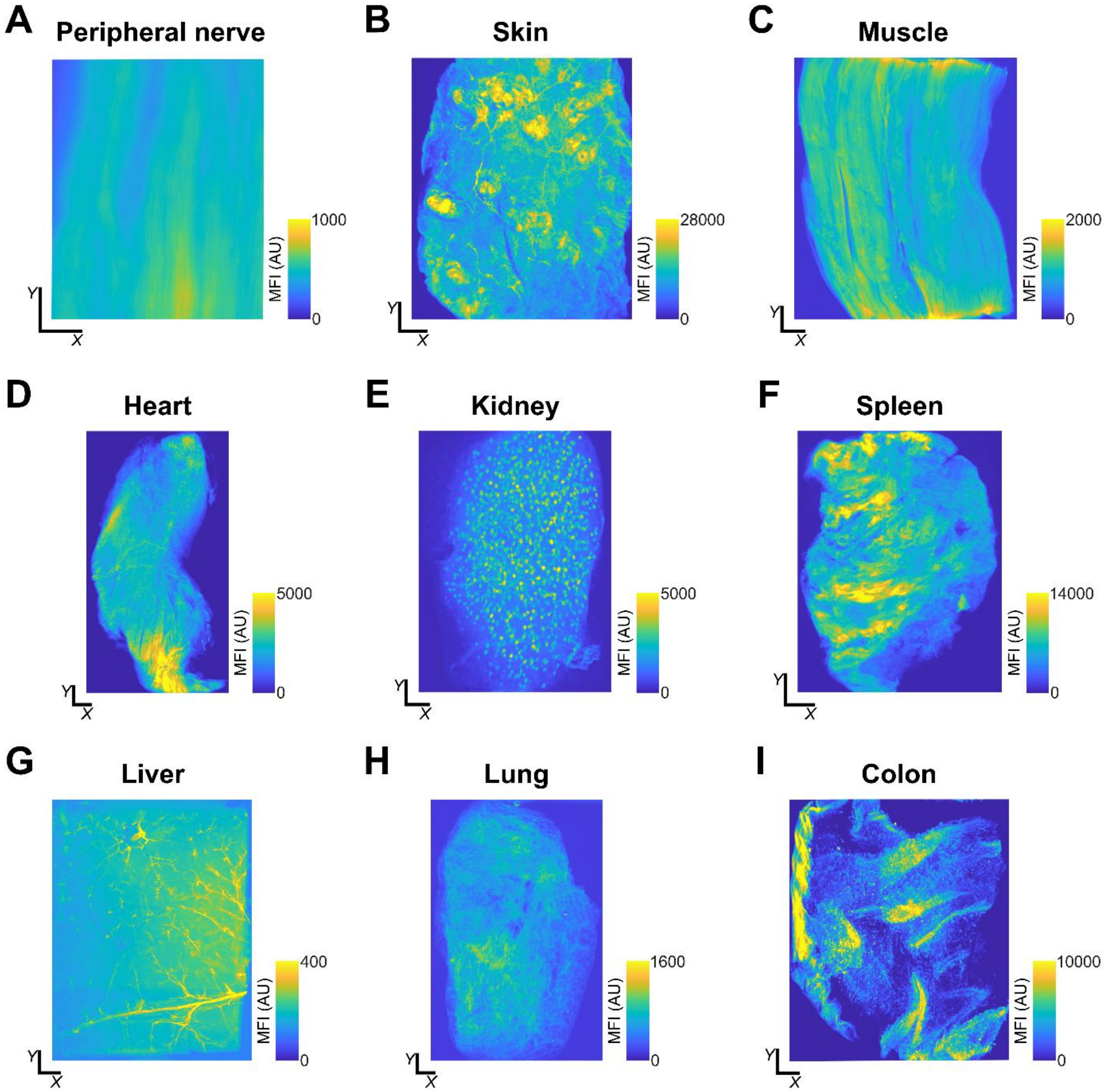

Fig. S7. Mean fluorescence intensity (MFI) projections of different archival human organs.
(**A-I**) MFI projections averaged over all planes in *z* and plotted as heatmaps in *xy*. Color scales indicate MFI ranges in arbitrary units (AU). Scale bars, 1 mm.

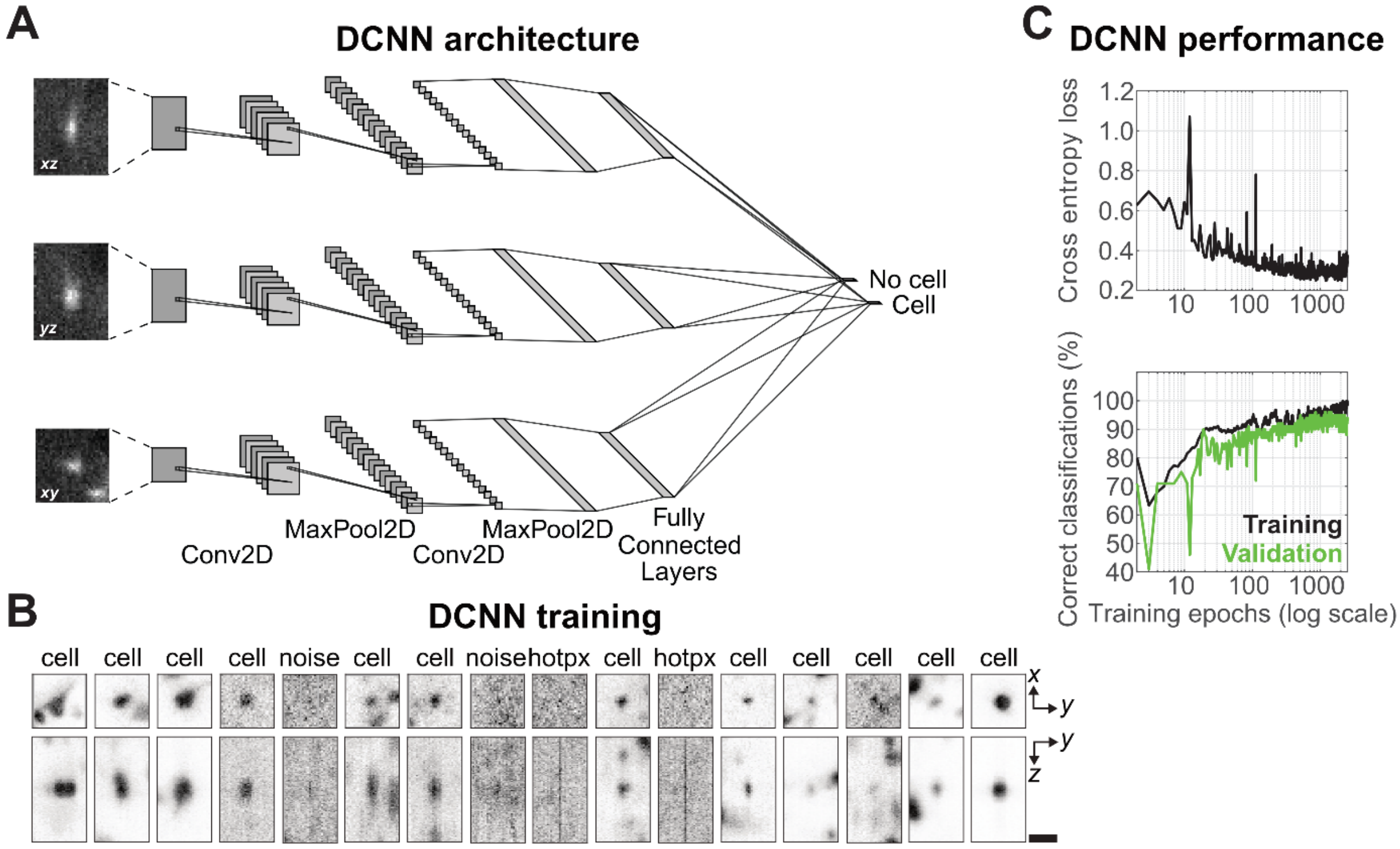

Fig. S8. DCNN architecture and performance.

(**A**) DCNN architecture for all three projections *xz, yz*, and *xy*. Each network consists of two convolutional (conv) 2D layers with ReLu functions, followed by a 2 x 2 max pooling (pool) 2D layer, two subsequent fully connected layers with ReLu functions, and a final layer with two neurons representing "cell" vs. "non-cell". The output of the three networks is combined to decide whether a cell candidate is a "cell" or "non-cell". (**B**) DCNN ground truth examples of the training input in different projections to classify "cell" vs. "non-cell" such as noise or hot pixels (hotpx) from the recording camera. For all excerpts, the full side lengths are 46.5 µm x 46.5 µm x 91 µm. Scale bar, 25 µm. (**C**) DCNN performance. Top: The cross entropy loss function decays during training and reaches a plateau after 1500 training epochs. Bottom: The percentage of correct classifications increases both for the training set (black) and for the validation set (green), and reaches a plateau of >90% after 1500 training epochs.

Table S1. Tested human organs and primary antibodies in different human tissue clearing methods.

	**iDISCO** (*13*)	**DIPCO** (*14*)	**MASH** (*15, 16*)	**SHANEL** (*17, 18*)	**aDISCO**	**OPTI Clear** (*19*)	**FAST Clear** (*20, 21*)	**CLARITY** (*22, 23*)	**SWITCH** (*24*) **SHIELD** (*25*)	**HIF** (*26*)	**ELAST** (*27*)	**CUBIC** (*28*)
**Human organs**	Brain	Lung<br>Colon<br>Pancreas<br>Ovary<br>Bladder<br>Head & neck	Brain<br>Prostate	Brain<br>Heart<br>Kidney<br>Spleen<br>Lung<br>Pancreas<br>Bone	**Brain**<br>**Spinal cord**<br>**Peripheral nerve**<br>**Skin**<br>**Muscle**<br>**Heart**<br>**Kidney**<br>**Spleen**<br>**Liver**<br>**Lung**<br>**Colon**	Brain	Brain<br>Spinal cord<br>Heart	Brain	Brain	Brain	Brain	Brain<br>Lung<br>Lymph node
**Primary anti-bodies**	Aβ<br>p-Tau	E-cad<br>N-cad<br>CD34<br>Vimentin	-	TH<br>NPY<br>MBP<br>IBA1<br>GFAP<br>Laminin<br>Coll4<br>SMA<br>Catalase<br>CK19	**NeuN**<br>**PV**<br>**TH**<br>**TPH2**<br>**NF**<br>**MBP**<br>**PGP9.5**<br>**IBA1**<br>**GFAP**<br>**PDXL**<br>**CD13**<br>**SMA**<br>**Laminin**<br>**Coll4**<br>**E-cad**<br>**N-cad**<br>**Nephrin**<br>**CD45**<br>**CK7**<br>**CK19**	Syn<br>TH<br>NF<br>IBA1<br>GFAP<br>ZO1<br>Tau<br>HLA DQ/ DR/DP	β-3-tubulin<br>NF<br>Vimentin	β-3-tubulin<br>HuC/D<br>PV<br>SST<br>TH<br>NF<br>MBP<br>IBA1<br>GFAP	NeuN<br>PV<br>TH<br>NPY<br>Calbin<br>Calret<br>NF<br>IBA1<br>GFAP<br>MMP13	MAP2<br>NF<br>GFAP	NeuN<br>MAP2<br>Syn<br>PSD95<br>PV<br>TH<br>NPY<br>Calbin<br>Calret<br>NF<br>MBP<br>GFAP	SMA<br>CK

Aβ, amyloid-beta; p-, phospho-; E-/N-cad, epithelial/neural cadherin; CD, cluster of differentiation; TH, tyrosine hydroxylase; NPY, neuropeptide Y; MBP, myelin basic protein; IBA1, ionizing calcium-binding adapter molecule 1; GFAP, glial fibrillary acidic protein; Coll4, collagen 4; SMA, smooth muscle actin; CK, cytokeratin; NeuN, neuronal nuclei; PV, parvalbumin; TPH2, tryptophane hydroxylase 2; NF, neurofilament; PGP9.5, protein gene product 5; PDXL, podocalyxin; Syn, synapsin; ZO1, zonula occludens 1; HLA, Human leukocyte antigen; HuC/D, human antigen C/D; SST, somatostatin; Calbin, calbindin; Calret, calretinin; MMP13, matrix metalloproteinase 13; MAP2, microtubule-associated protein 2; PSD95, postsynaptic density protein 95.

Table S2. Antibodies list.

Samples	Human organs	Antibody (species, clonality)	Source (order number)	LOT number	RRID	Dilution (start / final)	Staining time (3D histology)	Additional antigen retrieval
Fig. 5A	Peripheral nerve	AlexaFluor® 647 anti-Myelin Basic Protein (mouse, monoclonal)	Biolegend (808408)	B322585	AB_2814552	1:200 / 100	5 days	10% formic acid
Fig. S1B, S2B	Brain	Anti-Aminopeptidase N/CD13 (goat, polyclonal)	R&D systems (AF2335)	VRR0320081	AB_2227288	1:400 / 100	2 weeks	-
Fig. 4F, 5D, 5F, S6A	Spinal cord, heart, spleen	Anti-α-Actin, smooth muscle-Cy3™ (mouse, monoclonal)	Sigma Aldrich (C6198)	86029	AB_476856	1:800 / 200	2 weeks	- (30% formic acid for anti-CD45 co-staining)
Fig. 5I	Colon	Anti-Collagen 4 (goat, polyclonal)	Abcam (ab193951)	GR274637-5	AB_94799	1:1000 / 250	2 weeks	-
Fig. 5E	Kidney	Anti-E-Cadherin (rabbit, monoclonal)	Cell Signaling (3195S)	15	AB_2291471	1:800 / 200	2 weeks	-
Fig. S2C	Brain	Anti-GFAP (rabbit, polyclonal)	Agilent (Z0334)	00051304	AB_10013382	2D: 1:13000	2D only	2D: heated tris-borate-EDTA buffer
Fig. 5F	Spleen	Anti-Human CD45 (mouse, monoclonal)	Agilent (M0701)	41405417	AB_2314143	1:800 / 200	2 weeks	30% formic acid
Fig. S2C	Brain	Anti-Human CD68 (mouse, monoclonal)	Agilent (GA613)	41501009	AB_3712281	2D: 1:50	2D only	2D: heated tris-borate-EDTA buffer
Fig. 5E, S6B	Kidney	Anti-Human Nephrin (sheep, polyclonal)	R&D systems (AF4269)	ZMU0221031	AB_2154851	1:800 / 200	2 weeks	-
Fig. 4C, S1C, S3C	Brain	Anti-Human Podocalyxin (goat, polyclonal)	R&D systems (AF1658)	JLD0221051	AB_354920	1:400 / 100	2 weeks	10% formic acid
Fig. 4C, S2C	Brain	Anti-IBA1 (rabbit, polyclonal)	Fujifilm Wako (019-19741)	LER0547	AB_839504	1:800 / 200<br>2D: 1:1000	1 week	3D: -<br>2D: heated tris-borate-EDTA buffer
Fig. 5C	Muscle	Anti-Laminin (rabbit, polyclonal)	Sigma Aldrich (L9393)	VG3038968	AB_477163	1:800 / 200	2 weeks	-
Fig. 5D	Heart	Anti-N-Cadherin (rabbit, polyclonal)	Abcam (ab18203)	GR3404600-2	AB_444317	1:200 / 50	2 weeks	-
Fig. 4A, 6, 7, S2C, S3B, S4	Brain	Anti-NeuN (rabbit, polyclonal)	Merck Millipore (ABN78)	3692639	AB_10807945	1:800 / 200<br>2D: 1:400	2 weeks	3D: -<br>2D: heated tris-borate-EDTA buffer
Fig. 4E	Brain	Anti-Neurofilament pan-neuronal (mouse, monoclonal)	Biolegend (837801)	B279610	AB_2565383	1:800 / 200	2 weeks	10% formic acid
Fig. 4B	Brain	Anti-Parvalbumin	Swant (PV27)	PV27a_221215_200	AB_2631173	1:1000 / 250	2 weeks	10% formic acid

		(rabbit, polyclonal)						
Fig. 5B	Skin	Anti-PGP9.5 (rabbit, polyclonal)	Agilent (Z5116)	20074146	AB_2622233	1:400 / 100	2 weeks	-
Fig. 4D	Brain	Anti-Tryptophane Hydroxylase (mouse, monoclonal)	Merck Millipore (MAB5278)	3786041	AB_2207684	1:800 / 200	2 weeks	-
Fig. 3, S3A	Brain	Anti-Tyrosine Hydroxylase (rabbit, polyclonal)	Merck Millipore (AB152)	3705678	AB_390204	1:400 / 100	2 weeks	-
Fig. 4F	Spinal cord	Recombinant Alexa Fluor® 647 Anti-GFAP (rabbit, monoclonal)	Abcam (ab194325)	GR342668-1	N/A	1:2000 / 250	4 weeks	20% formic acid
Fig. 5H	Lung	Recombinant Anti-Cytokeratin 7 (rabbit, monoclonal)	Abcam (ab181598)	1015350-30	AB_2783822	1:2000 / 500	2 weeks	10% formic acid
Fig. 5G, S6C	Liver	Recombinant Anti-Cytokeratin 19 (rabbit, monoclonal)	Abcam (ab52625)	GR3384962-5	AB_2281020	1:800 / 200	2 weeks	-
Fig. 4C, S1C, S2B, S3C	Brain	Alexa Fluor® 647 AffiniPure Bovine Anti-Goat Secondary	Jackson Immuno Research (805-605-180)	156773	AB_2340885	1:400 / 100	2 weeks	-
Fig. 4D, 4E, 5F	Brain, spleen	Alexa Fluor® 647 AffiniPure Donkey Anti-Mouse Secondary	Jackson Immuno Research (715-605-150)	155989	AB_2340862	1:800 / 200	2 weeks	-
Fig. 5D	Heart	Alexa Fluor™ 647 Donkey anti-Rabbit Highly Cross-Adsorbed Secondary	Invitrogen (A-31573)	2359136	AB_2536183	1:200 / 50	2 weeks	-
Fig. 5E, S6B	Kidney	Alexa Fluor® 647 AffiniPure Donkey Anti-Sheep Secondary	Jackson Immuno Research (713-605-147)	2335729	AB_2340751	1:800 / 200	2 weeks	-
Fig. 5I, S1B	Colon, brain	Cy3™ AffiniPure Donkey Anti-Goat Secondary	Jackson Immuno Research (115-165-003)	149607	AB_2340813	1:800 / 200	2 weeks	-
Fig. 3, 4A-C, 5B-C, 5E, 5G-H, 6, 7, S3A-B, S4, S6C	Brain, skin, muscle, kidney, liver, lung	Cy3™ AffiniPure Donkey Anti-Rabbit Secondary	Jackson Immuno Research (711-165-152)	155995	AB_2307443	1:2000 / 500, 1:1000 / 250, 1:800 / 200, 1:400 / 100	2 weeks	-

Table S3. aDISCO clearing-induced tissue shrinkage of different human organs.

**Human organ**	Brain	Spinal cord	Peripheral nerve	Skin	Muscle	Heart	Kidney	Spleen	Liver	Lung	Colon
**Shrinkage (%)**	30 ± 0.0	12 ± 1.0	26 ± 8.0	49 ± 5.0	14 ± 1.0	32 ± 0.7	19 ± 0.7	41 ± 0.7	27 ± 10.6	30 ± 7.7	32 ± 4.9

Geometric mean of *x*- and *y*-dimensions ± with standard deviation.

Table S4. Characteristics of FCD patients and controls.

Pseudonymized patient ID	Age (years)	Gender	Brain localization	Diagnosis	2D evaluation throughout all layers	Clinical symptoms
2.2.	34	Male	Right frontal cortex	FCD IIb	No	Refractory epilepsy, impulse control disorder, mental retardation
2.3.	11	Male	Right frontal cortex	FCD IIb	No	Refractory epilepsy
2.5.	12	Male	Left parietal cortex	FCD IIb (restricted evaluation in 2D)	No	Refractory epilepsy
2.6.	17	Female	Right parietal cortex	FCD IIb	Yes	Refractory epilepsy, attention and lexical/orthographical impairment
2.7.	48	Male	Right parietal cortex	FCD IIb	Yes	Refractory epilepsy
2.8.	12	Female	Right temporal cortex	FCD IIa	Yes	Refractory epilepsy
2.10.	22	Female	Right temporal cortex	FCD IIa	No	Refractory epilepsy
1.2.	23	Male	Right frontal cortex	Control	N/A	Other (pseudonymized)
1.4.	11	Male	Right frontal cortex	Control	N/A	Other (pseudonymized)
1.5.	28	Male	Right frontal cortex	Control	N/A	Other (pseudonymized)
1.7.	45	Male	Right frontal cortex	Control	N/A	Other (pseudonymized)
1.8.	33	Female	Right frontal cortex	Control	N/A	Other (pseudonymized)
1.9.	40	Female	Right frontal cortex	Control	N/A	Other (pseudonymized)
1.10.	44	Male	Right frontal cortex	Control	N/A	Other (pseudonymized)

FCD, focal cortical dysplasia; N/A, not applicable.

Table S5. Summary of neuronal loss analyses in FCD patients.

FCD patient ID	#2	#3	#5	#6	#7	#8	#10
Number of visible lesional foci	1	2	11	25	2	5	16
Affected volume of brain (%)	1.25	0.41	2.65	3.67	0.06	3.98	3.32
Affected volume of cortex (%)	1.46	0.46	3.05	3.77	0.08	4.43	4.02
Affected optical slices (%)	4.7	48.1	89.2	54.4	14.8	53.9	75.2
Mean affected area fraction per optical slice (%)	0.73	0.27	4.00	2.47	0.05	2.73	5.02
Neuronal cell density (1000 per mm^3)	11.5	16.5	12.1	13.5	21.7	12.7	14.2
Neuronal cell density change compared to median density of controls (23.7 x 1000 per mm^3)	-51.3%	-30.3%	-48.9%	-43.1%	-8.0%	-46.1%	-40.1%

Table S6. Neuronal cell density in FCD patients and controls.

Neuronal cell density in FCD patients (1000 per mm^3)	11.5	16.5	12.1	13.5	21.7	12.7	14.2
Neuronal cell density in controls (1000 per mm^3)	13.0	30.5	27.1	23.7	19.7	26.9	13.3

Movie S1. Dopaminergic neurons in archival human midbrain.
3D rendering of an archival human midbrain sample, stained for dopaminergic neurons (anti-tyrosine hydroxylase) and imaged at 1x magnification. Zoom into the substantia nigra and switch to high-magnification imaging (12.6x) of a region-of-interest (ROI). Navigating through the ROI 3D rendering along the neurites, branching into a dense fiber network. Driving through the ROI 3D stack slice by slice along the *z*-axis, illustrating consistent antibody staining throughout the tissue block.

Movie S2. Neuronal tracing in archival human substantia nigra.
3D rendering of the substantia nigra from an archival human midbrain sample (region-of-interest as shown in Movie S1 and Fig. 3), stained for dopaminergic neurons (anti-tyrosine hydroxylase) and imaged at 12.6x magnification. The movie presents consecutive excerpts of raw mesoSPIM images (*xy*-planes) before increasing transparency to highlight the three-dimensional structure of the dendrites. Three traced dopaminergic neurons with their main branches are shown using color coding.

Movie S3. Cortical neurons in archival human brain.
3D rendering of an archival human brain cortex sample, stained for neurons (anti-NeuN) and imaged at 1.25x magnification. Zoom into the cortex and switch to high-magnification imaging (12.6x) of a region-of-interest (ROI), highlighting layers I-IV. Driving through the ROI 3D stack slice by slice along the *z*-axis, illustrating the large number of human cortical neurons in this sample, with distinct architecture of pyramidal and granular neurons.

Movie S4. Microglia and vessels in archival human hippocampus.
3D rendering of an archival human hippocampus sample, stained for microglia (cyan; anti-IBA1) and vessels (magenta; anti-podocalyxin), and imaged at 0.8x magnification. Zoom into the center of the sample and switch to high-magnification imaging (12.6x) of a region-of-interest (ROI). Navigating through the ROI 3D rendering, highlighting microglia morphology with their processes close or attached to blood vessels. Zoom-out and switch back to 3D rendering at 0.8x magnification, going back to the start position.

Movie S5. Nerve fibers in archival human skin.
3D rendering of an archival human skin sample, stained for nerve fibers (anti-PGP9.5) and imaged at 1.6x magnification. Zoom into the dermis and navigating along the long sensory nerve fibers up to a Pacinian corpuscle. Zoom-out and navigating to the epidermis. Zoom-in, turn, and switch to 4x magnification imaging of a region-of-interest (ROI) in the transversal view. Navigating through the ROI 3D rendering up to the thin sensory nerve fibers, which innervate the epidermis, and zoom-out.

Movie S6. Glomeruli and tubules in archival human kidney.
3D rendering of an archival human kidney sample, stained for glomeruli (blue; anti-nephrin) and for tubules (cyan; anti-E-cadherin), and imaged at 1x magnification. Zoom into the center of the sample up to 3x magnification. Driving through the 3D stack slice by slice along the *z*-axis, illustrating the large number of glomeruli in this kidney sample. Further zoom into one glomerulus and switch to high-magnification imaging (12.6x), highlighting glomerulus morphology with Bowman capsule, outgoing proximal tubule, and surrounding distal tubule by driving

through the region-of-interest (ROI) 3D stack slice by slice along the *z*-axis. Switch to 3D rendering of the ROI and zoom-out. Switch back to 3D rendering at 1x magnification and further zoom-out up to the starting position.

Movie S7. Detected cortical neurons in archival human brain.

Stitched and 4x-downsampled 3D stack of an archival human brain cortex sample, stained for neurons (anti-NeuN) and imaged at 4x magnification. Driving through the 3D stack slice by slice along the *z*-axis, while zooming into a region-of-interest (ROI), highlighting cortical layers III and IV with pyramidal and granular neurons, respectively. Overlay of the raw 3D stack with detected neurons (yellow) and driving through the 3D stack slice by slice along the *z*-axis to illustrate the overlap between originally stained and detected neurons.
